# GNSS Spoofing Detection Method Based on the Pseudorange Difference Between Epochs for Single-Antenna Receivers

**DOI:** 10.3390/s26206516

**Published:** 2026-10-14

**Authors:** Shimiao Chen, Feisheng Li, Zehao Zhang, Qiyang Zhang, Hailong Wu, Xiabin Dong

**Affiliations:** Taiyuan Satellite Launch Center, Taiyuan 030000, China

**Keywords:** GNSS spoofing detection, antispoofing, pseudorange difference, multi-epoch, single-antenna

## Abstract

Spoofing attacks against the Global Navigation Satellite System (GNSS) are difficult to perceive and highly destructive, and they therefore constitute a serious security threat to GNSS services. Although numerous countermeasures have been reported, those exploiting the direction of the incoming signal generally rely on antenna arrays, on several cooperating receivers, or on a single antenna kept in motion, all of which raise the hardware cost and the implementation complexity. Motivated by this limitation, this article develops a spoofing detection scheme for single-antenna receivers that operates on the pseudorange difference between epochs. The key idea is that this epoch-wise difference can be predicted with high fidelity from ephemeris data and successive positioning solutions, so that under authentic conditions the measured and the predicted differences agree in a statistical sense. Once spoofing is present, the geometric and kinematic mismatch between the true satellite and the spoofer, together with the pull-off manoeuvre of the counterfeit signal, destroys that agreement and thereby exposes the attack. Because no assumption is imposed on the number of transmitting sources, the scheme is in principle able to reveal spoofing radiated from several directions at once. Monte Carlo trials are carried out to quantify how the individual design parameters govern detection capability, and the scheme is then verified on a public dataset as well as on a dataset collected by the authors. The outcome confirms that spoofing arriving from different directions is reliably identified with one antenna only, which relaxes the antenna requirement imposed by direction-based methods for multi-agent spoofing; detection is further shown to be more favourable when the receiver is moving or when the spoofer pulls off at a high rate. The detector is most responsive while the counterfeit observables are being pulled away from the authentic ones, and the margin it retains once the attack has settled into a steady state depends on how faithfully the attacker tracks the motion of the target, so that an absolute or geometry-based integrity monitor is the natural complement wherever that margin becomes narrow.

## 1. Introduction

Reliance on the Global Navigation Satellite System (GNSS) keeps deepening across both defence and civil sectors, and the robustness of the service has become a matter of corresponding concern. A number of successful attacks reported in the recent literature [1,2,3] illustrate the point: spoofing now ranks among the factors capable of compromising the safe and stable operation of GNSS. What sets it apart from jamming is that it is at once harder to notice and more damaging in its consequences.

Currently, many spoofing detection methods have been proposed. In the early stages of research on spoofing detection methods, scholars conducted spoofing detection by searching for signal features that changed after a receiver was spoofed. Due to the inability of spoofing signals to perfectly simulate the power of real signals, the addition of spoofing can cause changes in signal power [4], carrier-to-noise ratio [5], etc. In the capture and tracking stage, under the covert spoofing strategy, the spoofing signal needs to enter the tracking loop and slowly increase the power and change phase, which can cause misalignment between the real code and the spoofing code phases and the appearance of multiple correlation peaks [6]. Detectors of this family, which react to the distortions listed above, are the ones most frequently deployed in practice and cope well with unsophisticated attacks.

The combination of inertial navigation and GNSS can achieve the purpose of spoofing detection by checking the consistency of trajectory, acceleration, position, and other characteristics. Broumandan et al. [7] compared the trajectory estimated by the receiver with the trajectory obtained by the Inertial Navigation System. Kwon et al. [8] compared the accelerations calculated using accelerometers and receivers. Many scholars use encryption and authentication techniques in cryptography to encrypt navigation messages or spread spectrum chips, and then the receiver detects spoofing through encryption verification. According to the different encryption authentication methods, this method can be divided into Navigation Message Authentication [9,10] and Spreading Code Authentication [11]. Any countermeasure resting on cryptographic authentication presupposes a change to the signal-in-space itself, and such a change is unlikely to materialise quickly.

Another line of research exploits the fact that a spoofer and the genuine constellation are seen from different directions, together with the observables that this angular discrepancy modulates. He et al. [12] reported a dual-antenna scheme in which the frequency difference of arrival across a fixed baseline is predicted from carrier phase measurements and navigation data. Authentic signals keep the predicted and the measured values aligned, whereas a spoofer, whose geometry and motion relative to the antennas differ from those of the satellite, breaks that alignment and is thus exposed even when several spoofing sources act together.

Working with an antenna array, Chen et al. [13] recovered the intersection angle between two directions of arrival (IA-DOA) from the known baseline geometry of the array elements and then submitted the measured and the predicted angles to a Generalized Likelihood Ratio Test (GLRT), which allows spoofing radiated from several directions to be recognised. A different route was taken by Li et al. [14], who drove a single antenna in a reciprocating vertical motion and inferred the incidence angle of each signal from the amplitude ripple that this motion imprints upon it. A related effort in [15] kept a single antenna in steady rotation, the circular baseline traced by the element then supplying the geometry needed to recover the IA-DOA of signals arriving from more than one direction. Liu et al. [16] instead formed the pseudorange double difference over two epochs to solve for the receiver coordinates, and judged the authenticity of the signals by checking whether that solution agrees with the one delivered by conventional least squares.

Among the strategies surveyed above, those reasoning about where a signal comes from are the least easy to defeat and currently offer the strongest guarantees. In prior work [17], an intersection-angle-based (IA-DOA) detection method was developed for single-antenna receivers by forming spatial–temporal pseudorange double differences across satellite pairs. However, because that method strictly relies on the receiver’s spatial displacement to recover angular geometry, it inevitably loses sensitivity when the receiver is in a stationary state.

To address this limitation, the present article retains the single-antenna premise of [17] but dispenses with the inter-satellite angular formulation, operating directly on the epoch-wise pseudorange difference on a per-satellite basis. This scalar formulation provides three key benefits: (1) detection is performed per channel rather than constellation-wide, isolating contaminated signals; (2) the pseudorange pull-off itself serves as detection evidence, restoring the ability to flag advanced spoofing against stationary receivers; and (3) an IMM-KF stage is introduced to robustly condition the trajectory estimates driving the prediction model.

In a clean environment the measured and the modelled pseudorange difference coincide statistically; both the angular offset of the counterfeit signal and the error carried by the spoofed position solution erode that coincidence. Detection capability is first characterised through Monte Carlo trials and subsequently confirmed on the public TEXBAT recordings and on data gathered by the authors. Since one antenna suffices irrespective of whether the attack originates from a single transmitter or from several, the hardware premise usually attached to direction-aware detectors is substantially relaxed.

Stated in that way, the contribution invites being read as yet another spoofing detector. The question pursued here is more elementary and is not tied to any particular countermeasure: how can the temporal consistency of the GNSS observables themselves be exploited to recognise, autonomously and without external assistance, that the positioning, navigation and timing (PNT) solution a receiver is delivering has lost its trustworthiness, especially in the regime where spatial diversity is unavailable, that is, where no antenna array, no cooperating receiver and no deliberate motion of the antenna can be relied upon? Casting the problem in terms of trustworthiness rather than in terms of a specific attack signature carries a concrete consequence: the evidence has to be drawn from quantities the receiver already produces, and the verdict has to be issued per channel, because under a partial attack only some of the tracked signals are counterfeit while the rest remain genuine. A second message follows from the same framing and turns out to be equally important: temporal consistency is violated most severely while the counterfeit observables are being dragged away from the authentic ones, and it is only gradually restored once the spoofer settles into a steady state. The detector is therefore at its strongest during the transition, and the margin it retains afterwards depends on how faithfully the attacker manages to track the motion of the target. Section 5 documents this behaviour on public recordings and identifies, for the narrow-margin case, the complementary monitoring that restores full coverage.

It is equally important to state what this work is not. Resilient PNT is generally understood as the ability of a navigation system to do more than detect an attack: it must also maintain service, degrade gracefully, isolate the faulty source and restore a trustworthy solution, and the chain usually invoked runs from nominal PNT through attack onset and detection, then to degraded or untrusted PNT, then to mitigation or isolation, and finally to the recovery and reacquisition of trusted PNT. The detector developed below occupies a single link of that chain. It is a detection and integrity-monitoring component that decides whether the observables of one channel remain temporally consistent with what the receiver’s own state predicts; it does not isolate the counterfeit signals, and it neither maintains nor restores a navigation solution, so no claim of that kind is advanced here. What it delivers is an integrity flag marking the affected channel as untrusted, and the subsequent isolation, the switch to an alternative source and the reacquisition of a trusted solution belong to the layer above. The operational setting envisaged is accordingly a specific one: a mobile platform that has to keep navigating with a single antenna and without external aiding, such as an unmanned aerial vehicle or a road vehicle, for which an undetected spoofing attack translates directly into a safety-critical loss of position and time. Stationary installations are deliberately not put forward as the primary case, for a reason that the experimental section makes explicit.

The remainder of the article is organised as follows. Section 2 derives the underlying principle and the associated theory. Section 3 introduces the detector built on the pseudorange difference. Section 4 examines, through software simulation, the parameters governing detection capability. Section 5 reports the validation and the performance assessment carried out on real datasets. Section 6 discusses the portability of the method across receiver platforms and constellations and states its limitations. Section 7 closes the article and outlines directions for further study.

## 2. System Model

This section reveals an estimation method for pseudorange differences based on navigation information and proposes an optimisation method for estimating pseudorange differences.

### 2.1. Observations of Pseudorange Difference

For the *i*-th satellite tracked at epoch *t*, the pseudorange delivered by the receiver admits the following model:

(1) $${\overset{⌢}{\rho }}_{i}\left(t\right)=r_{i}\left(t\right)+c\cdot \delta t_{u}\left(t\right)-c\cdot \delta t_{i}\left(t\right)+I_{i}\left(t\right)+\zeta_{i}\left(t\right)+\varepsilon \left(t\right),$$

in which $r_{i}\left(t\right)$ denotes the geometric range separating the *i*-th satellite from the receiver at epoch *t*, *c* is the speed of light, $\delta t_{u}\left(t\right)$ and $\delta t_{i}\left(t\right)$ are the clock offsets of the receiver and of the *i*-th satellite at that epoch, $I_{i}\left(t\right)$ and $\zeta_{i}\left(t\right)$ collect the ionospheric and the tropospheric propagation delays, and ε(t) lumps together unmodelled effects, modelling inaccuracies and measurement noise.

Let $X_{i}\left(t\right)$ and $x_{r}\left(t\right)$ stand for the positions of the *i*-th satellite and of the receiver at epoch *t*, both expressed in the Earth-centred Earth-fixed frame. The range $r_{i}\left(t\right)$ then follows as:

(2) $$r_{i}\left(t\right)=∥X_{i}\left(t\right)-x_{r}\left(t\right)∥.$$

Subtracting the measurements taken from the same satellite at two neighbouring epochs yields the observation of the pseudorange difference:

(3) $$\Delta {\overset{⌢}{\rho }}_{i}\left(k\right)={\overset{⌢}{\rho }}_{i}\left(k\right)-{\overset{⌢}{\rho }}_{i}\left(k-1\right)=r_{i}\left(k\right)-r_{i}\left(k-1\right)+\eta \left(k\right).$$

Here ${\overset{⌢}{\rho }}_{i}\left(k\right)$ and ${\overset{⌢}{\rho }}_{i}\left(k-1\right)$ are the pseudoranges output by the receiver at epoch *k* and at the preceding epoch, and η(k) collects the high-order and unmodelled terms together with the measurement noise.

This approach of slow variation within a short time instant eliminates the constant integer ambiguities and most of the common-mode errors from the satellite clock bias, the ephemeris, the multipath, and the atmosphere [18]. In addition, the common receiver clock drift increment $c\cdot \Delta \delta t_{u}\left(k\right)$ shared across all tracked satellites is directly removed by subtracting the constellation-wide mean difference $\frac{1}{M}\sum_{m=1}^{M}\left(\Delta {\overset{⌢}{\rho }}_{m}\left(k\right)-\Delta {\bar{\rho }}_{m}\left(k\right)\right)$ (where *M* is the number of tracked satellites) or estimated through the navigation solution.

### 2.2. Predictions of Pseudorange Difference

As detailed below, the prediction of pseudorange difference can be calculated through ephemeris and receiver movement trajectory.

The Time Differenced Carrier Phase (TDCP) technique differences the carrier phase of one satellite over two consecutive epochs and, in doing so, cancels the integer ambiguity along with the bulk of the ionospheric and tropospheric contributions, which benefits both positioning accuracy and reliability [19]. The time-differenced pseudorange examined below is constructed along the same lines. Referring to Figure 1, over one epoch interval the satellite travels from A to E while the receiver travels from B to C; writing $r_{i}\left(k\right)$ and $r_{i}\left(k-1\right)$ for the satellite-to-receiver ranges at the two epochs leads to:

(4) $$\begin{array}{cc} \Delta r_{i}\left(k\right) & =r_{i}\left(k\right)-r_{i}\left(k-1\right)=e_{i}\left(k-1\right)\cdot \Delta X_{i}\left(k\right)\\ \; & -e_{i}\left(k-1\right)\cdot \Delta x_{r}\left(k\right)+\eta \left(k\right), \end{array}$$

where $e_{i}\left(k-1\right)$ is the unit vector from the receiver to the satellite at epoch k−1, $\Delta X_{i}\left(k\right)=X_{i}\left(k\right)-X_{i}\left(k-1\right)$ is the satellite position variation, $\Delta x_{r}\left(k\right)=x_{r}\left(k\right)-x_{r}\left(k-1\right)$ is the receiver position variation, and η(k) is the high-order small term.

Due to the significant geometric distance between the satellite and the receiver and the slight movement distance of the receiver at adjacent times, the variation of the pseudorange can be expressed as:

(5) $$\Delta {\bar{\rho }}_{i}\left(k\right)=e_{i}\left(k-1\right)\cdot \Delta X_{i}\left(k\right)-e_{i}\left(k-1\right)\cdot \Delta x_{r}\left(k\right).$$

The two satellite positions $X_{i}\left(k\right)$ and $X_{i}\left(k-1\right)$ follow from the broadcast ephemeris, whereas the two receiver positions $x_{r}\left(k\right)$ and $x_{r}\left(k-1\right)$ are taken from the navigation solution.

Denoting by $\Delta {\bar{\rho }}_{i,\mathrm{sat}}\left(k\right)=e_{i}\left(k-1\right)\cdot \Delta X_{i}\left(k\right)$ the portion of the pseudorange difference attributable to the displacement of the navigation satellite, and by $\Delta {\bar{\rho }}_{i,\mathrm{rcv}}\left(k\right)=-e_{i}\left(k-1\right)\cdot \Delta x_{r}\left(k\right)$ the portion attributable to the displacement of the receiver, (5) takes the compact form:

(6) $$\Delta {\bar{\rho }}_{i}\left(k\right)=\Delta {\bar{\rho }}_{i,\mathrm{sat}}\left(k\right)+\Delta {\bar{\rho }}_{i,\mathrm{rcv}}\left(k\right).$$

In summary, the prediction of pseudorange difference can be obtained based on the position information of the multi-epoch receiver and the ephemeris information.

### 2.3. Optimisation Methods for Predictions

The error between the pseudorange observation value and the satellite navigation positioning result affects the prediction of the pseudorange difference. The Kalman filter is used to optimise the pseudorange observation value and satellite navigation and positioning results to reduce the influence of error on the prediction value of the pseudorange difference.

Using a single-motion filtering model can reduce robustness because receivers are generally used in automobiles, ships, drones, etc. Therefore, the Interactive Multiple Model (IMM) algorithm of the Constant Velocity (CV) motion model and the Constant Turn (CT) motion model is used to describe the motion of the system. The IMM algorithm uses multiple motion models to describe a constantly changing system, with each model corresponding to a filtering algorithm. The result of fusing multiple models based on model probability is used as the output at each moment [20,21].

The critical problem in the IMM algorithm is the selection of the filtering algorithm. In this article, the measurements feeding the IMM-KF are the position and velocity outputs from the receiver’s navigation solution, and the state-space observation equation is linear, which does not require nonlinear conversion. Therefore, the Kalman filter is used for filtering calculation directly. In summary, to reduce the impact of errors on the prediction of pseudorange differences, this article uses the IMM-KF algorithm to filter the position information. It should be noted that the IMM-KF algorithm is a filtering method used to improve detection performance in this article and is not the focus of this study. If there are better optimization methods for positioning results in the future, IMM-KF can be replaced.

## 3. Proposed Method

A detector is now constructed around the agreement between the measured and the modelled pseudorange difference. Two mechanisms undermine that agreement once an attack is under way: the counterfeit signal generally arrives from a direction other than the true one, and the attacker deliberately manipulates the evolution of the pseudorange in order to steer the victim. Either mechanism separates the observation from the prediction, and it is this separation that the detector exploits.

### 3.1. Principle of Consistency Check

Formed from raw pseudorange measurements, the observed pseudorange difference carries the genuine kinematics of the satellite–receiver pair, while its predicted counterpart is reconstructed independently from the receiver position and the broadcast ephemeris. Section 2 established that these two quantities coincide statistically as long as the tracked signal is authentic, their residual being zero-mean Gaussian in that case. Following (6), the residual reads:

(7) $$e_{i}\left(k\right)=\Delta {\overset{⌢}{\rho }}_{i}\left(k\right)-\Delta {\bar{\rho }}_{i}\left(k\right),$$

where $e_{i}\left(k\right)$ is the residual that includes the unmodeled effects, modelling errors, multipath errors, and measurement errors. Rapid changes in multipath can lead to false detection. It is necessary to use Fault Detection and Exclusion (FDE) [22] techniques to verify the measurement set consistency and to eventually exclude the blunder. Only verified measurements should be used to build the pseudorange differences.

For the convenience of subsequent analysis, the residual can be divided into two parts: $e_{i,\mathrm{sat}}\left(k\right)$ and $e_{i,\mathrm{rcv}}\left(k\right)$, where $e_{i,\mathrm{sat}}\left(k\right)$ represents the residual of the pseudorange difference caused by the satellite’s motion and $e_{i,\mathrm{rcv}}\left(k\right)$ represents the residual of the pseudorange difference caused by the receiver’s motion. When the receiver is not spoofed, both $e_{i,\mathrm{sat}}\left(k\right)$ and $e_{i,\mathrm{rcv}}\left(k\right)$ conform to the normal distribution with zero mean.

An attacker mounts the deception either by relaying authentic signals or by synthesising signals that embed falsified navigation data. Whichever objective is pursued, a displacement in position or a shift in time, the pseudorange difference conveyed by the counterfeit signal need not match the one conveyed by the genuine signal. Two circumstances make that mismatch surface in the residual: the counterfeit signal arriving along a direction other than the true one, and the attacker being unable to pin down the victim position accurately.

When the receiver is spoofed, the residual between the observation and the prediction of the pseudorange difference of the spoofing signal will not conform to the normal distribution with zero mean value. The following is a detailed analysis of different spoofing cases. Table 1 establishes four spoofing cases based on two criteria: whether the direction of the spoofing signal is the same as the direction of the real signal and whether the receiver’s solution position is the same as the real position, and lists possible types of spoofing.

Figure 2, Figure 3, Figure 4 and Figure 5 show the pseudorange differences between the real and the spoofing signals in the four cases. $\Delta x^{r}\left(k\right)$ (black) denotes the true movement trajectory of the receiver from the ${\left(k-1\right)}^{\mathrm{th}}$ to the $k^{\mathrm{th}}$ epoch, while $\Delta x^{s}\left(k\right)$ (red) denotes the trajectory computed from the spoofing-induced positioning result over the same interval. $\Delta {\overset{⌢}{\rho }}_{i,\mathrm{rcv}}\left(k\right)$ (black) is the observed pseudorange difference caused by the receiver motion, that is, the pseudorange-difference observation due to the receiver’s movement. $\Delta {\bar{\rho }}_{i,\mathrm{rcv}}\left(k\right)$ (red) is the predicted pseudorange difference caused by the receiver motion, computed from the ephemeris data and the positioning result. It should be noted that both generated and retransmitted spoofing signals can well simulate the pseudorange difference caused by satellite motion, that is, $e_{i,\mathrm{sat}}\left(k\right)=\Delta {\overset{⌢}{\rho }}_{i,\mathrm{sat}}\left(k\right)-\Delta {\bar{\rho }}_{i,\mathrm{sat}}\left(k\right)$ obeys a zero-mean normal distribution.

The epoch-wise pseudorange difference exploited by the detector is the superposition of three contributions whose physical origins differ, and although Figure 2, Figure 3, Figure 4 and Figure 5 draw them with a common set of vectors, the three can be told apart as follows. The first is the contribution of the receiver displacement between the two consecutive epochs, $\Delta {\bar{\rho }}_{i,\mathrm{rcv}}\left(k\right)=-e_{i}\left(k-1\right)\cdot \Delta x_{r}\left(k\right)$; it reflects a *position error* whenever the position solutions entering $\Delta x_{r}$ are falsified, and it is carried in the figures by the two displacement vectors $\Delta x^{r}\left(k\right)$ and $\Delta x^{s}\left(k\right)$. The second is the contribution of the satellite displacement projected onto the line of sight, $\Delta {\bar{\rho }}_{i,\mathrm{sat}}\left(k\right)=e_{i}\left(k-1\right)\cdot \Delta X_{i}\left(k\right)$; it depends on the *signal direction*$e_{i}$, so that a counterfeit signal radiated from a direction other than the true one modifies this unit vector and thereby corrupts the corresponding term. The third is the offset $\Delta \rho_{i,\mathrm{error}}\left(k\right)$ that a *time-spoofing* operation injects by advancing or retarding the phase of the counterfeit signal. In a clean environment the third term vanishes and the first two are reproduced with high fidelity by the prediction built from the ephemeris and the navigation solution, so that the residual $e_{i}\left(k\right)$ remains zero-mean; spoofing makes the third term non-zero and, through the falsified position solution, perturbs the first term as well, which is precisely what the detector senses.

In Case 1, the spoofing location result is the same as the real location result, and the signal sent by the spoofer is the same as the real signal, as shown in Figure 2. At this time, the pseudorange difference caused by the receiver motion satisfies $\Delta {\overset{⌢}{\rho }}_{i,\mathrm{rcv}}\left(k\right)=\Delta {\bar{\rho }}_{i,\mathrm{rcv}}\left(k\right)$, that is, the residual $e_{i,\mathrm{rcv}}\left(k\right)$ conforms to a zero-mean normal distribution. The fact that the spoofing location result coincides with the real position indicates that the spoofing type at this time is time spoofing. Time spoofing is achieved by modifying the phase of the spoofing signal, which introduces an unreasonable change in the pseudorange difference, denoted as $\Delta \rho_{i,\mathrm{error}}\left(k\right)$. The residual of the pseudorange difference in Case 1 is represented as:

(8) $$e_{i,\mathrm{spoof}}\left(k\right)=e_{i,\mathrm{sat}}\left(k\right)+e_{i,\mathrm{rcv}}\left(k\right)+\Delta \rho_{i,\mathrm{error}}\left(k\right)+\xi \left(k\right),$$

where $e_{i,\mathrm{spoof}}\left(k\right)∼N\left(\Delta \rho_{i,\mathrm{error}}\left(k\right),\sigma^{2}\right)$ and $\Delta \rho_{i,\mathrm{error}}\left(k\right)\neq 0$.

In Case 2, the spoofing location result is different from the real location result, and the signal sent by the spoofer is the same as the real signal, as shown in Figure 3. At this time, the pseudorange difference caused by the receiver motion satisfies $\Delta {\overset{⌢}{\rho }}_{i,\mathrm{rcv}}\left(k\right)\neq \Delta {\bar{\rho }}_{i,\mathrm{rcv}}\left(k\right)$, so the residual $e_{i,\mathrm{rcv}}\left(k\right)$ conforms to a normal distribution with a non-zero mean. If the spoofing signal simultaneously engages in time spoofing, it introduces an unreasonable change in the pseudorange difference, denoted as $\Delta \rho_{i,\mathrm{error}}\left(k\right)$. The residual of the pseudorange difference in Case 2 is given by (8).

In Case 3, the spoofing location result is the same as the real location result, and the signal sent by the spoofer is different from the real signal, as shown in Figure 4. At this time, the pseudorange difference caused by the receiver motion satisfies $\Delta {\overset{⌢}{\rho }}_{i,\mathrm{rcv}}\left(k\right)\neq \Delta {\bar{\rho }}_{i,\mathrm{rcv}}\left(k\right)$, so the residual $e_{i,\mathrm{rcv}}\left(k\right)$ conforms to a normal distribution with a non-zero mean. The fact that the spoofing location coincides with the real position indicates that the spoofing type at this time is time spoofing. Time spoofing is achieved by modifying the phase of the spoofing signal, which introduces an unreasonable change in the pseudorange difference, denoted as $\Delta \rho_{i,\mathrm{error}}\left(k\right)$. The residual of the pseudorange difference in Case 3 is given by (8).

In Case 4, the spoofing location result is different from the real location result, and the signal sent by the spoofer is different from the real signal, as shown in Figure 5. At this time, the pseudorange difference caused by the receiver motion satisfies $\Delta {\overset{⌢}{\rho }}_{i,\mathrm{rcv}}\left(k\right)\neq \Delta {\bar{\rho }}_{i,\mathrm{rcv}}\left(k\right)$, so the residual $e_{i,\mathrm{rcv}}\left(k\right)$ conforms to a normal distribution with a non-zero mean. If the spoofing signal simultaneously engages in time spoofing, it introduces an unreasonable change in the pseudorange difference, denoted as $\Delta \rho_{i,\mathrm{error}}\left(k\right)$. The residual of the pseudorange difference in Case 4 is given by (8).

In the above four spoofing cases, when and only when $\Delta \rho_{i,\mathrm{error}}\left(k\right)=0$, $e_{i}\left(k\right)$ conforms to the normal distribution with a mean of zero. Therefore, it can be considered that spoofing signals will disrupt the consistency between the observation and prediction values of pseudorange difference. The hypothesis test is established as follows:

(9) $$\left\{\begin{array}{c} H_{0}:E\left[e_{i}\left(k\right)\right]=0,\\ H_{1}:E\left[e_{i}\left(k\right)\right]\neq 0. \end{array}\right.$$

The null hypothesis $H_{0}$ corresponds to a clean environment in which measurement and model agree statistically, whereas $H_{1}$ signals the presence of a counterfeit signal. A decision can therefore be reached by accumulating, over successive epochs, the discrepancy separating the observed from the predicted pseudorange difference.

It should be noted that the above analysis in this article does not assume that the spoofer is in a stationary state [23] or only contains one transmitting antenna [24]. Therefore, the above methods can detect mobile and multi-antenna spoofers.

### 3.2. Optimisation of the Detection Method

Although the pseudorange observation has been optimised through the Kalman filter, the observation of the pseudorange difference still has errors. It is necessary to suppress the measurement error of pseudorange difference to achieve better performance. Therefore, we use the average method to optimise pseudorange observations and improve spoofing detection performance.

The detection variables for the mean method are defined as follows:

(10) $$T_{i}={\left(\frac{1}{K}\sum_{j=1}^{K}e_{i}\left(k-j\right)\right)}^{2}/\left(\frac{\sigma^{2}}{K}\right),$$

where *K* is the number of average values.

According to the analysis in the previous section, $e_{i}\left(k\right)$ conforms to the normal distribution, assuming that the mean value of $e_{i}\left(k\right)$ is μ and the standard deviation is σ. Because the low-frequency dynamic errors and common biases are largely tracked and mitigated by the IMM-KF filtering stage and the differencing operation, the successive difference residuals $e_{i}$ can be reasonably treated as independent and identically distributed (i.i.d.) random variables. Then the mean value of the variable $\frac{1}{K}\sum_{j=1}^{K}e_{i}\left(k-j\right)$ is μ and the standard deviation is $\sigma /\sqrt{K}$. Therefore, using the average method reduces the negative impact of measurement errors and can improve the performance of spoofing detection methods. Furthermore, establish hypothesis testing as follows:

(11) $$\left\{\begin{array}{c} H_{0}:T_{i}∼\chi^{2}\left(1\right),\\ H_{1}:T_{i}∼\chi_{1}^{2}\left(\lambda \right). \end{array}\right.$$

Under the null hypothesis $H_{0}$, $T_{i}$ follows a central Chi-squared distribution with 1 degree of freedom, denoted as $\chi^{2}\left(1\right)$; under the alternative hypothesis $H_{1}$, $T_{i}$ follows a non-central Chi-squared distribution with 1 degree of freedom and non-centrality parameter $\lambda =K\mu^{2}/\sigma^{2}$, which is determined by the spoofing characteristics. The probability density functions under the null hypothesis and alternative hypothesis are:

(12) $$\left\{\begin{array}{cc} f_{H_{0}}\left(x\right)=\frac{1}{\sqrt{2\pi x}}e^{-x/2}, & x>0,\\ f_{H_{1}}\left(x\right)=\frac{1}{\sqrt{2\pi x}}e^{-(x+\lambda )/2}I_{0}\left(\sqrt{\lambda x}\right), & x>0, \end{array}\right.$$

in which $I_{0}\left(\cdot \right)$ is the zero-order modified Bessel function of the first kind.

Furthermore, establish a generalised likelihood ratio test:

(13) $$\Lambda =\frac{\max_{H_{1}}f_{H_{1}}\left(T_{i}\right)}{\max_{H_{0}}f_{H_{0}}\left(T_{i}\right)}\underset{H_{1}}{\overset{H_{0}}{<}}\gamma ,$$

where γ denotes the decision threshold. Fixing γ through the Neyman–Pearson criterion pins down the false alarm probability $P_{fa}$ and the detection probability $P_{d}$ attained at that threshold:

(14) $$\left\{\begin{array}{c} P_{fa}=\int_{\gamma }^{+\infty }f_{H_{0}}\left(x\right)dx,\\ P_{d}=\int_{\gamma }^{+\infty }f_{H_{1}}\left(x\right)dx. \end{array}\right.$$

The preceding derivation leads to the detection procedure sketched in Figure 6, whose individual stages are described below:

**Step 1: Data Collection and Optimization**

1. Recover the satellite coordinates at the epochs of interest from the broadcast ephemeris, read the pseudorange measurements produced while tracking the navigation signal, and solve for the receiver position.
2. Optimise the positioning results using IMM-KF.

**Step 2: Calculation of the Pseudorange Difference in Multiple Epochs**

1. Calculate the observations of multi-epoch pseudorange difference based on pseudorange observations according to Formula (3);
2. Based on ephemeris information and positioning results, calculate the predictions of multi-epoch pseudorange difference according to Formula (5).

**Step 3: Likelihood Ratio Test**

1. Determine the monitoring threshold based on the set detection and false alarm probability.
2. Calculate the detection variable according to the Formula (10). If it is less than the threshold, the source signal of the current pseudorange is considered a real signal; otherwise, it is considered a spoofing signal. It should be noted that each visible satellite corresponds to a detection variable, and the threshold setting is independent of the number of satellites.

## 4. Performance Analysis

The behaviour of the proposed detector is now examined by Monte Carlo trials. Running the algorithm with and without the IMM-KF establishes what the filtering stage contributes. Four quantities are then varied in turn, namely the pseudorange measurement noise, the output rate of the receiver, the speed at which the receiver travels and the window length of the averaging operation, so that the sensitivity of the detector to each of them can be assessed.

### 4.1. Simulation Environment Settings

Each trial proceeds in four stages. The trajectory followed by the receiver, the coordinates of the spoofers and the interval over which spoofing is active are configured first, and the ephemeris of the constellation visible over that interval is exported from STK. Pseudoranges are then synthesised from the geometry linking satellites, spoofers and receiver, and corrupted with noise. A navigation solution is subsequently computed from those pseudoranges. The detection statistics $T_{i}$ are finally evaluated with the proposed method, the threshold being fixed by the Neyman–Pearson criterion so that detection capability can be quantified.

Table 2 collects the configuration adopted for the trials; unless a deviation is explicitly stated, every experiment reported below relies on these values.

The ground truth trajectory used in the trials is plotted in Figure 7. Departing from the origin of the local frame, the receiver heads north at 5 m/s, executes a right turn at 0.1 rad/s once 100 s have elapsed, reverses into a left turn at the same angular rate at 150 s, and travels along a straight line during the remaining intervals.

ECEF coordinates of the eight satellites visible throughout the trials are listed in Table 3. Spoofing is produced by repeaters: the first spoofer relays the signals whose PRNs are 30, 7, 28 and 17, while the second relays those whose PRNs are 11, 8, 13 and 1, so that the two sources radiate distinct subsets of the constellation.

Monte Carlo trials supply the detection statistics $T_{i}$ of every satellite under each configuration examined, which is what makes a thorough performance assessment possible. Reporting the Receiver Operating Characteristic (ROC) for all of them would, however, multiply the number of curves and consume a disproportionate amount of space.

The Neyman–Pearson criterion is a minimum error rate criterion used to determine the decision threshold of a binary classifier. This criterion aims to minimise the total error rate of false positives and false negatives. Neyman–Pearson criterion determines the detection threshold, and the detection probability under the optimal threshold can be considered the optimal detection probability. The higher the optimal detection probability, the better the performance of the detection algorithm. Therefore, this article chooses the detection probability under the Neyman–Pearson criterion as the evaluation criterion for algorithm performance.

### 4.2. Results with IMM-KF

To analyse the impact of the optimisation method proposed in this article on detection performance, Figure 8 compares the detection performance obtained without and with IMM-KF optimisation. The horizontal axis of the figure is the receiver channel, numbered 1 to 8, and the vertical axis is the optimal detection probability of that channel. The two curves therefore report the same eight channels under the two configurations: the blue squares correspond to the case without the optimisation method and the orange circles to the case with it.

Two effects of the IMM-KF stage are visible in the figure. The first is a rise of the overall level: averaged over the eight channels, the optimal detection probability is 97.30% without the optimisation method and 98.92% with it, an improvement of 1.62 percentage points. The second is a reduction in the channel-to-channel spread: without the optimisation method the individual channels range from 96.29% to 98.31%, whereas with it all eight channels lie between 98.89% and 98.96%, so that the weakest channel alone gains about 2.6 percentage points. In summary, the IMM-KF optimisation method proposed in this article can effectively reduce the impact of errors and improve the performance of detection algorithms.

### 4.3. Results with Different Pseudorange Observation Errors

The pseudorange measurement noise is swept over $\sigma_{\rho }=1$ to 10 m in steps of 1 m, that is, all ten integer values are simulated. Figure 9 reports the outcome: panel (a) gives, for each setting of $\sigma_{\rho }$, the optimal detection probability of the eight individual channels, while panel (b) gives their arithmetic mean, so that point *k* of panel (b) is the average of the eight curves at point *k* of panel (a). Detection capability is seen to deteriorate monotonically as the noise level grows, the mean of the eight channels falling from 99.19% at $\sigma_{\rho }=1$m to 98.85% at $\sigma_{\rho }=10$m. The variation is nevertheless small—less than 0.35 percentage points across the whole decade of noise levels examined—which indicates that the method tolerates a substantial degradation of the pseudorange measurements without losing its detection capability.

Reason analysis: Noisier pseudoranges translate directly into a noisier epoch-wise pseudorange difference, which blurs the separation between the two hypotheses and hence degrades detection. The method proposed in this article focuses on the signal of each channel separately, which can achieve spoofing detection for a single channel. There may be differences in the offset speed of spoofing from different channels, which means that the pseudorange offset of different spoofing signals is different. The larger the offset of the pseudorange difference caused by the spoofing with a fast pseudorange offset, the easier it is to be detected. Therefore, the simulation results show a clear relationship between the detection probability and the channel under different standard deviation values.

### 4.4. Results with Different Numbers of Samples Set for the Mean Method

The averaging window is swept from 100 up to 1000 samples in steps of 100. Figure 10 reports the outcome in the same two-panel layout: Figure 10a gives the optimal detection probability reached by the individual channels for each window length, and Figure 10b gives their mean, point *k* of Figure 10b again being the average of the eight curves at point *k* of Figure 10a. The mean rises overall with the window length, from 98.67% at 100 samples to 98.92% at 1000 samples, and reaches its maximum of 98.95% at 700 samples; the rise is not strictly monotonic, mild dips of the order of 0.05 percentage points occurring between 400 and 500 samples and again between 700 and 900 samples. Beyond roughly 600 samples the additional gain is small, so the choice of the averaging window trades a modest improvement in detection probability against the response time that a longer window necessarily introduces.

Reason analysis: Increasing the sample size of the average method can reduce the impact of error on detection performance, because the averaging suppresses the zero-mean measurement noise while the systematic component induced by the spoofing signal is retained. The benefit is nevertheless quickly exhausted, since the mean curve is essentially flat once a few hundred samples are used, and enlarging the window further only lengthens the interval over which the statistic is accumulated. Because in the initial stage of spoofing the weight of the pseudorange difference caused by the spoofing signal is still small, a longer accumulation interval also delays the moment at which the detection threshold is crossed, and hence delays the alarm. In summary, increasing the sample size of the average method can improve detection performance, but a moderate window is to be preferred.

### 4.5. Results with Different Data Refresh Frequencies of the Receiver

The output rate of the receiver is swept over 1 to 10 Hz in steps of 1 Hz. Figure 11 reports the outcome in the same two-panel layout, Figure 11a giving the optimal detection probability attained by the individual channels at each rate and Figure 11b their mean. Detection is seen to improve monotonically with the output rate: the mean of the eight channels rises from 97.54% at 1 Hz to 99.72% at 10 Hz, a gain of more than two percentage points, and no saturation of the improvement is observed anywhere in the range examined.

Reason analysis: A higher output rate delivers more measurements within the same observation window, and the larger sample set that the detector then averages over attenuates the influence of the errors, which lifts detection capability.

### 4.6. Results with Different Speeds of the Receiver

The speed of the receiver is swept over 1 to 10 m/s in steps of 1 m/s. Figure 12 reports the outcome in the same two-panel layout, with Figure 12a giving the optimal detection probability of the individual channels at each speed and Figure 12b their mean. The dominant feature is the step between the two slowest settings: at 1 m/s the mean of the eight channels is only 94.71%, whereas at 2 m/s it already reaches 98.75%, a gain of about four percentage points. Beyond 2 m/s the mean remains within the narrow band of 98.74% to 98.93% and no further systematic trend is visible, so it is the near-static regime, rather than the high-speed regime, that limits the detector.

Reason analysis: The faster the receiver travels, the longer the baseline it sweeps between two consecutive epochs. That baseline is deduced from the difference of two navigation solutions, whose absolute error does not depend on speed, so a longer baseline carries a smaller relative error. Since this displacement is one of the inputs feeding the prediction of the pseudorange difference, its refinement propagates into a more faithful prediction and, in turn, into a stronger detection capability. This mechanism accounts for the abrupt gain observed between 1 and 2 m/s; once the relative error of the baseline has become small compared with the remaining error sources, a further increase in the speed brings no additional benefit, which is why the mean curve is essentially flat above 2 m/s.

Taken together, the five parametric studies reported above support a small number of conclusions rather than five independent ones. First, detection is governed mainly by the temporal separation between the evolution of the authentic pseudorange and that of the counterfeit one: whatever widens that separation, a faster pull-off rate above all, raises the statistic and shortens the time to alarm, whereas a slow pull-off leaves the two evolutions nearly parallel and therefore difficult to tell apart. Second, motion of the receiver strengthens observability, because the displacement swept between consecutive epochs is one of the inputs of the predicted difference, and a longer swept baseline carries a smaller relative error. That benefit is almost entirely realised as soon as the receiver leaves the near-static regime: the mean detection probability jumps by about four percentage points between 1 and 2 m/s and then remains flat, so it is the near-static regime, rather than the high-speed regime, that limits the detector, and it is the caveat that any stationary installation must carry. Third, the remaining factors, namely the measurement noise level, the length of the averaging window and the data refresh rate, act upon the sensitivity of the test rather than upon its validity: at a fixed false-alarm probability they shift the detection probability that is attained, but they leave the ordering of the cases unchanged. The same holds for the IMM-KF stage, which refines the prediction fed into the statistic without altering the detection principle. It is these three statements, and not the individual parameter sweeps, that the experiments on recorded data are meant to confirm.

## 5. Experimental Results and Performance Evaluation

To confirm that the proposed detector works in practice and to quantify how well it works, recordings acquired under spoofing are replayed through a software receiver and the resulting observables are submitted to the detection algorithm. Because the internal architecture of a receiver conditions what any spoofing detector can achieve, the processing platform is stated explicitly below.

The intermediate frequency samples are processed with the FGI-GSRx software receiver [25], whose source code is publicly available at https://github.com/nlsfi/FGI-GSRx (accessed on 11 October 2026), and its raw observables and PVT solutions, both delivered at 10 Hz, are stored for the subsequent detection stage. Two collections are exercised: the openly distributed TEXBAT recordings, and a set of recordings captured by the authors under multi-agent spoofing.

### 5.1. Review of the TEXBAT

Released by the University of Texas as a common benchmark for spoofing countermeasures, the Texas Spoofing Test Battery (TEXBAT) comprises two clean recordings and eight recordings acquired under attack, labelled ds1 to ds8 [26,27]. Each attack pursues either a timing objective or a positioning objective, which is what the spoofing type reported for the individual scenarios denotes.

The dataset ds1 simulates that the spoofer can physically access the receiver’s antenna, block the real signal from entering the target receiver, and directly replace the receiver’s signal with spoofing, which is unrealistic. Therefore, ds1 will not be analysed in subsequent experiments. Table 4 summarises the basic parameters for each scenario in the spoofing datasets. The offset speed of time spoofing is approximately 13.3 ns/s (4 m/s equivalent). The offset speed of position spoofing is approximately 4 m/s.

### 5.2. Introduction to Multi-Agent Spoofing Datasets

The measurement campaign is described first. As illustrated in Figure 13, the testbed comprises two spoofing sources, a receiving antenna and an intermediate frequency acquisition unit; the two sources jointly emulate a multi-agent attacker, while the acquisition unit, fed by the antenna, records everything that reaches it.

The sources are labelled A and B and split the counterfeit constellation between them: A radiates PRNs 10, 12 and 15, whereas B radiates PRNs 18, 23 and 24. Within the local frame, whose coordinates are expressed in metres, the antenna occupies the origin while A and B sit at (0, 2, 0.5) and (0, −2, 0.5), respectively. Combining the two motion states of the receiver with the two spoofing objectives produces the four scenarios summarised in Table 5.

The offset speed of time spoofing is 5 ns/s (1.5 m/s equivalent), and after the completion of the time offset, it will remain in an offset state. The result of location spoofing is a circular motion with a radius of 80 m and an angular velocity of 0.2rad/s, with an offset line velocity of 16 m/s.

### 5.3. Analysis of Experimental Results

This section verifies the spoofing detection algorithm on the dataset TEXBAT and MASD and analyses its performance. First, the datasets containing spoofing are processed to obtain the changing trend of detection variables $T_{i}$. Then, according to the different threshold values, the ROC for different sample numbers is obtained, which is convenient for analysing detection performance. The algorithm proposed in this article can perform spoofing detection on signals from a certain channel separately, so signals from different channels correspond to an ROC curve.

Comparing the TEXBAT with the MASD, it was found that for the TEXBAT, the value of the detection variable T after offset completion is relatively low, which means it is challenging to achieve spoofing detection at this time; For the MASD, the value of the detection variable T remains high after offset completion, which means that spoofing detection can be achieved at this time. The reasons for the above phenomenon are analysed below.

Within TEXBAT the counterfeit and the authentic signals are brought into agreement to within a few metres at the point where the victim antenna stands, because the attacker there is granted timely and precise knowledge of how the target moves and can correct the transmission accordingly. Therefore, after the spoofing offset is completed, the observation and prediction of the pseudorange difference calculated based on the spoofing are consistent, and the detection method will fail. This indicates that the proposed velocity/difference-based detector is most sensitive to the dynamic pull-off stage; once steady-state spoofing is established without velocity bias, the detector should be coupled with absolute geometry-based integrity checks. This asymmetry between the transient and the steady state is not a peculiarity of a single recording: it is a property of any detector that reasons on temporal consistency, and it therefore delimits the envelope within which the present method is expected to operate on its own, rather than indicating a defect of the formulation. In most practical situations an attacker cannot obtain real-time and accurate information about how the target moves, so a residual discrepancy between the observation and the prediction of the pseudorange difference survives once the pull-off has been completed, and the method proposed in this article continues to detect the spoofing. Full consistency in the steady state thus presupposes an unusually capable attacker and represents the demanding end of the range rather than the typical case. It should be noted that the detection variable T can be obtained based on the signals in different channels, which means that spoofing detection of a single signal can be achieved.

Before the ROC panels are read, the physical meaning of their two axes should be stated explicitly. The horizontal axis is the false alarm probability $P_{fa}$, that is, the probability that a non-spoofed channel is nevertheless declared spoofed, whereas the vertical axis is the detection probability $P_{d}$, that is, the probability that a spoofed channel is correctly declared spoofed; both are defined in (14). Each curve is traced by sweeping the decision threshold γ of (13): raising γ lowers $P_{fa}$ but also lowers $P_{d}$, while lowering γ raises $P_{d}$ at the cost of a higher $P_{fa}$. In operation γ is fixed in advance from the tolerated false alarm probability through the Neyman–Pearson criterion, and the detection probability actually attained is the ordinate of the ROC curve at that abscissa. Because $T_{i}$ follows a central $\chi^{2}$ distribution with one degree of freedom under $H_{0}$, the threshold follows directly from that distribution; Table 6 lists the values of γ corresponding to the false alarm probabilities most frequently adopted.

**Table 6:** Decision threshold γ of the Neyman–Pearson test as a function of the tolerated false alarm probability $P_{fa}$, evaluated from the central $\chi^{2}$ distribution with one degree of freedom of (11). The detection probability attained at each threshold is read from the ROC curves of Figure 14 at the corresponding $P_{fa}$.

False Alarm Probability $P_{\mathrm{fa}}$	Decision Threshold γ
$5\times 10^{-2}$	3.84
$10^{-2}$	6.63
$10^{-3}$	10.83
$10^{-4}$	15.14

To analyse the impact of sample size on detection performance, this article calculates the AUC (Area Under Curve) corresponding to the ROC curve. We use the average AUC value under different channels as an evaluation indicator to facilitate the analysis of detection performance under different datasets and sample sizes. The average value of AUC is shown in Figure 15. As the number of samples increases, AUC shows an increasing trend, which means increasing the number of samples will improve detection performance. However, when the sample size is large, the benefits of increasing the sample size decrease and even lead to performance degradation (such as DS6). This is consistent with the simulation analysis results.

To analyse the impact of IMM-KF on detection performance, Figure 16 shows the average AUC value when using or not using IMM-KF to optimize simulation position solutions with a sample size of 300. From the figure, it can be seen that the use of IMM-KF has improved detection performance to a certain extent.

The absolute error of the difference in positioning results between epochs when the receiver is in dynamic and static states is the same, and the displacement distance when the receiver is in a dynamic state is greater than that when it is in a static state. By comparing the derived formulas for the pseudorange difference’s prediction of multiple epochs, it is found that the relative error of the difference in positioning result between epochs when the receiver is in the dynamic state is lower than that when it is in the static state. Furthermore, the accuracy of the pseudorange difference’s prediction of the receiver in the dynamic state is higher than that in the static state. The pseudorange difference’s prediction in the detection variable is influenced by the pseudorange difference’s prediction between epochs, and the mean of the detection variable in the dynamic state is lower than that in the static state.

The above analysis indicates that the spoofing detection method proposed in this article performs better when the receiver is in motion. By analysing the AUC of the time spoofing dataset when the receiver is in the static (ds2, ds3, ds7, ds8, and MASD1) and moving (ds5 and MASD3) states, it can be found that the spoofing detection performance is better in the moving state. By analysing the AUC of the location spoofing dataset when the receiver is in the static (ds4 and MASD2) and moving (ds6 and MASD4) states, it can be found that the spoofing detection performance is better in the moving state. In summary, it can be concluded that the spoofing detection method performs better when the receiver is in motion. The above observation results are consistent with the theoretical analysis results.

The detection method proposed in this article uses pseudorange difference as the detection variable. The faster the offset speed of the spoofing signal, the greater the difference between the observation and prediction of the pseudorange difference of the spoofing. This means that the faster the offset speed of the spoofing, the better the spoofing detection performance. This section introduces the offset speed of time spoofing and location spoofing in the dataset TEXBAT and MASD. The speed of time offset and position offset in the TEXBAT dataset is the same. So, the detection performance under time spoofing and location spoofing should be similar in both stationary and moving situations. The experimental results are consistent with theoretical analysis by comparing the AUC of time spoofing and location spoofing under the same motion state in the TEXBAT.

The offset speed of time spoofing in the MASD dataset is smaller than the offset speed of location spoofing, so the performance of location spoofing detection should be better than that under time spoofing. The experimental results are consistent with theoretical analysis by comparing the AUC of time spoofing and position spoofing under the same motion state in the MASD dataset.

Taken together, the outcomes obtained on TEXBAT and on MASD show that attacks mounted by several cooperating sources are reliably exposed by the proposed detector across the whole span of transmit powers, spoofing objectives and receiver motion states considered here. It is worth noting that while the proposed epoch-wise pseudorange difference detector successfully resolves the blind spot of advanced spoofing under static conditions, its detection sensitivity in stationary scenarios is naturally lower than that under kinematic conditions due to the lack of dynamic baseline expansion. Compared with the dual-satellite IA-DOA scheme in [17], which benefits from strong geometric constraints during motion, the present per-channel method trades peak sensitivity for standalone channel isolation and static operability. In practical engineering deployments, the two approaches can either be switched adaptively based on the receiver’s motion state or fused collaboratively to achieve comprehensive protection across diverse operational environments.

The same outcomes are perhaps most usefully summarised in terms of the operating states that a PNT system traverses, rather than in terms of algorithm parameters. (i) In the nominal state, before any counterfeit signal is present, the statistic remains inside the distribution implied by the measurement noise, and the false-alarm behaviour is set entirely by the threshold; Table 6 lists the thresholds corresponding to the false-alarm probabilities of practical interest. (ii) During attack onset and pull-off the statistic leaves that distribution, and the rate at which it does so is governed by the pull-off rate, by the motion of the receiver and by the measurement noise, exactly as the parametric study of Section 4 anticipated. That rate is what fixes the time to alarm: a faster pull-off drives the statistic across the threshold sooner, whereas a slower pull-off, and likewise a longer averaging window, postpones the crossing. No absolute delay is quoted, because the instant at which the attack begins is not annotated with sufficient precision in either recording. (iii) Once the pull-off is complete and the attacker has settled into a steady state, the two collections diverge: on MASD the statistic stays well clear of the threshold, whereas on TEXBAT, where the attacker is granted precise and timely knowledge of the target motion, it returns towards its nominal distribution and the margin narrows. How much margin survives therefore depends on how faithfully the attacker tracks the target motion, and the recordings show that a usable margin is preserved as soon as that knowledge is less than exact. (iv) The fourth state, recovery, in which the counterfeit signals disappear or are excluded and the statistic is expected to fall back to its nominal distribution so that the receiver can declare PNT trustworthy again, is not covered by the recordings used here: neither TEXBAT nor MASD contains an interval following the termination of the attack. Characterising that state, and in particular the time the statistic needs to return below the threshold and the confidence with which trust can then be re-declared, is left for future work.

## 6. Discussion and Limitations

The detector developed in the preceding sections draws on three standard outputs of a navigation receiver—the pseudorange measurements, the broadcast ephemeris and the position solutions—and on no quantity that is tied to a particular receiver architecture. It requires neither access to the correlator outputs, nor carrier-phase observables, nor additional radio-frequency channels, and it can therefore be hosted, in principle, on any receiver that provides the three quantities above. The present validation was carried out with the publicly available FGI-GSRx software receiver so that the results can be reproduced by others, but this choice reflects reproducibility rather than a dependence of the method on that platform. It should nevertheless be recognised that different receiver platforms differ in their pseudorange measurement noise, their multipath mitigation capability and their clock stability. All of these enter the detection statistic through the noise level of the epoch-wise pseudorange difference, so that the same decision threshold will translate into a slightly different detection probability from one platform to another; quantifying that dependence across a range of commercial receivers and output rates is a natural extension for future work.

A second point that deserves comment is the applicability to multiple constellations. The detection statistic is built on the epoch-wise pseudorange difference of a single channel, and the decision is taken independently for each tracked satellite, the threshold fixed through the Neyman–Pearson criterion being independent of the number of satellites. A GPS channel, a BeiDou channel and a Galileo channel can therefore be tested in parallel without interfering with one another, and a mixed-constellation receiver is accommodated without any modification to the algorithm, provided that the broadcast ephemeris of the corresponding system is available for reconstructing the predicted pseudorange difference. What does differ from one system to another is the pseudorange noise level, the ephemeris accuracy and, under attack, the pull-off rate that the spoofer applies to each system; these differences are expected to show up in the detection probability rather than in the validity of the method, and a systematic comparison of the detection performance across constellations is left for future work.

A third point concerns the place of this detector within a resilient PNT architecture, and it is here that the scope of the contribution has to be stated most carefully. A resilient PNT chain is usually described as running from nominal operation through attack onset and detection, then to degraded or untrusted PNT, then to mitigation or isolation, and finally to the recovery and reacquisition of a trusted solution. The method proposed in this article supplies one link of that chain and one link only. It decides, per channel and from quantities a receiver already produces, whether the observables remain temporally consistent with the state the receiver predicts for itself; it does not isolate the counterfeit signals, it does not maintain the navigation service or degrade it gracefully, and it does not restore a trusted solution. It is therefore a detection and integrity-monitoring component rather than a complete resilient PNT scheme, and no claim of the latter kind is advanced here. What it returns is an integrity flag raised on the affected channel, and the isolation of that channel, the switch to an alternative source, the graceful degradation of the service and the eventual reacquisition of a trusted solution belong to the layers above. Two consequences follow for the intended application. Because the statistic is driven by the transient during which the counterfeit observables are dragged away from the authentic ones, the detector is best matched to users whose own motion makes that transient observable, that is, to mobile platforms such as unmanned aerial vehicles and road vehicles that must keep navigating with a single antenna and without external aiding; for a stationary installation the margin is narrower and the detector ought to be paired with an absolute or geometry-based integrity monitor. For the same reason, an attack that has already reached a steady state under a very capable attacker leaves a narrower margin, and a deployment that is meant to be resilient is best complemented by a monitor that does not rest on temporal consistency alone; this is a division of labour between complementary monitors rather than a deficiency of the present one.

## 7. Conclusions

This work leads to four conclusions.

1. **The method and its validated range.** The detector serves a single-antenna receiver and runs on the pseudorange difference between epochs. A prediction model driven by ephemeris data and position solutions supplies the expected difference; pairing that prediction with the corresponding measurement yields a detection statistic, evaluated independently for every tracked channel. Monte Carlo trials characterised the performance, and the detector was then exercised on the TEXBAT and MASD recordings. Spoofing was identified across the whole range of transmit powers, spoofing objectives and receiver motion states covered by those recordings. The margin is wider when the receiver is moving than when it is stationary, and wider still when the attacker pulls off quickly. Because nothing in the formulation requires the counterfeit signals to share a common direction of arrival, attacks mounted from several directions remain within reach of the detector.
2. **Where the margin is narrowest.** The detector is at its strongest while the counterfeit observables are being dragged away from the authentic ones. Once the attacker has settled into a steady state, the surviving margin depends on how faithfully that attacker tracks the target’s motion. On TEXBAT, where the attacker is granted an accurate live estimate of that motion, the residual returns towards its nominal distribution and the margin becomes narrow; the recordings where the attacker’s knowledge is imperfect keep a usable margin throughout. This is what any monitor that reasons on temporal consistency must do, not an artefact of one dataset. Where the margin becomes narrow, the detector calls for an absolute or geometry-based integrity check to complement it.
3. **The effect of receiver motion on detection.** What governs detection is how far the authentic and counterfeit pseudorange evolutions separate in time. Receiver motion widens that separation, lengthening the baseline swept between consecutive epochs. The detector is consequently markedly stronger on a moving platform than on a stationary one. The intended setting is a mobile platform that must keep navigating with a single antenna and without external aiding, such as an unmanned aerial vehicle or a road vehicle; a stationary installation is deliberately not put forward as the primary case. The IA-DOA detector of [17] relies on spatial-temporal angular constraints between satellite pairs. The present per-channel formulation trades some peak sensitivity for channel isolation and for operability without motion. The two schemes can therefore be selected adaptively or combined according to the receiver’s motion state.
4. **What the detector does not do.** The method supplies one link of a resilient PNT chain: it detects the loss of trustworthiness and raises an integrity flag on the affected channel. Isolating the counterfeit signals, keeping or gracefully degrading the service, and restoring a trusted solution all sit in the layers above; this work makes no such claim. The recordings used in this work do not cover the recovery state, in which the statistic returns to its nominal distribution and trust can be re-declared; that case stays open for future study.

## Figures and Tables

**Figure 1:**
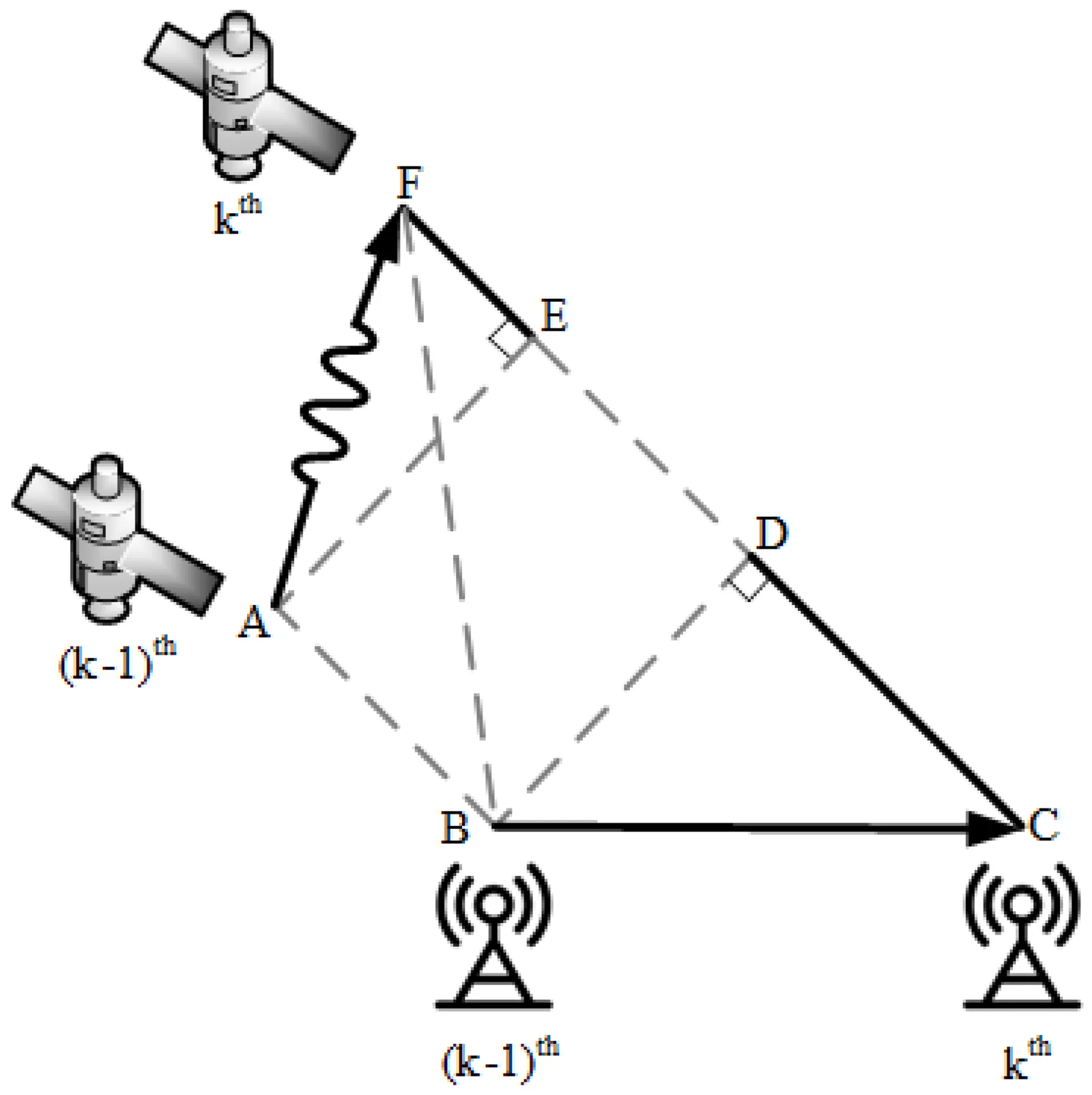
Pseudorange difference model. In general, AF is much greater than BC.

**Figure 2:**
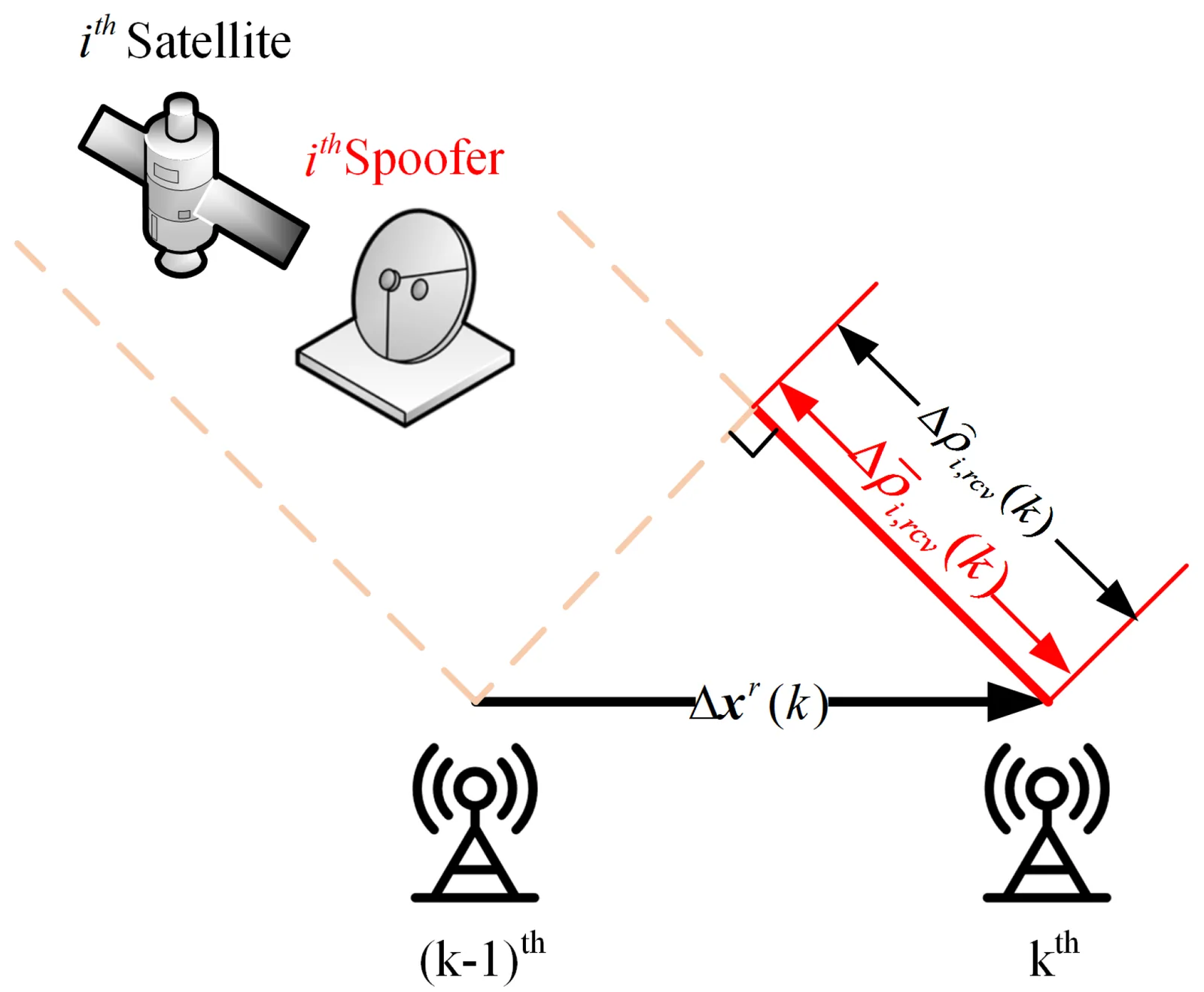
Analysis of the pseudorange difference in Case 1.

**Figure 3:**
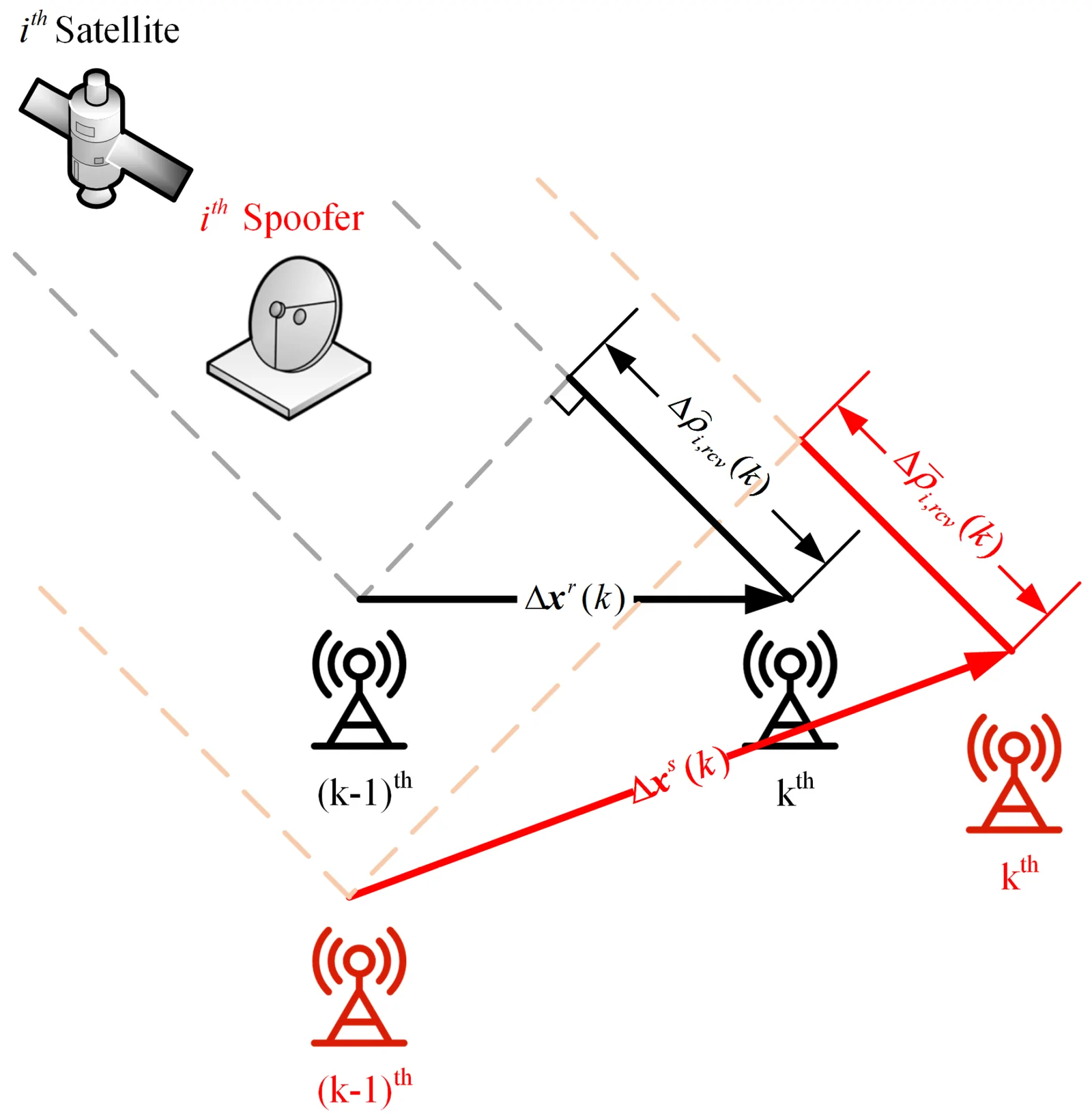
Analysis of the pseudorange difference in Case 2.

**Figure 4:**
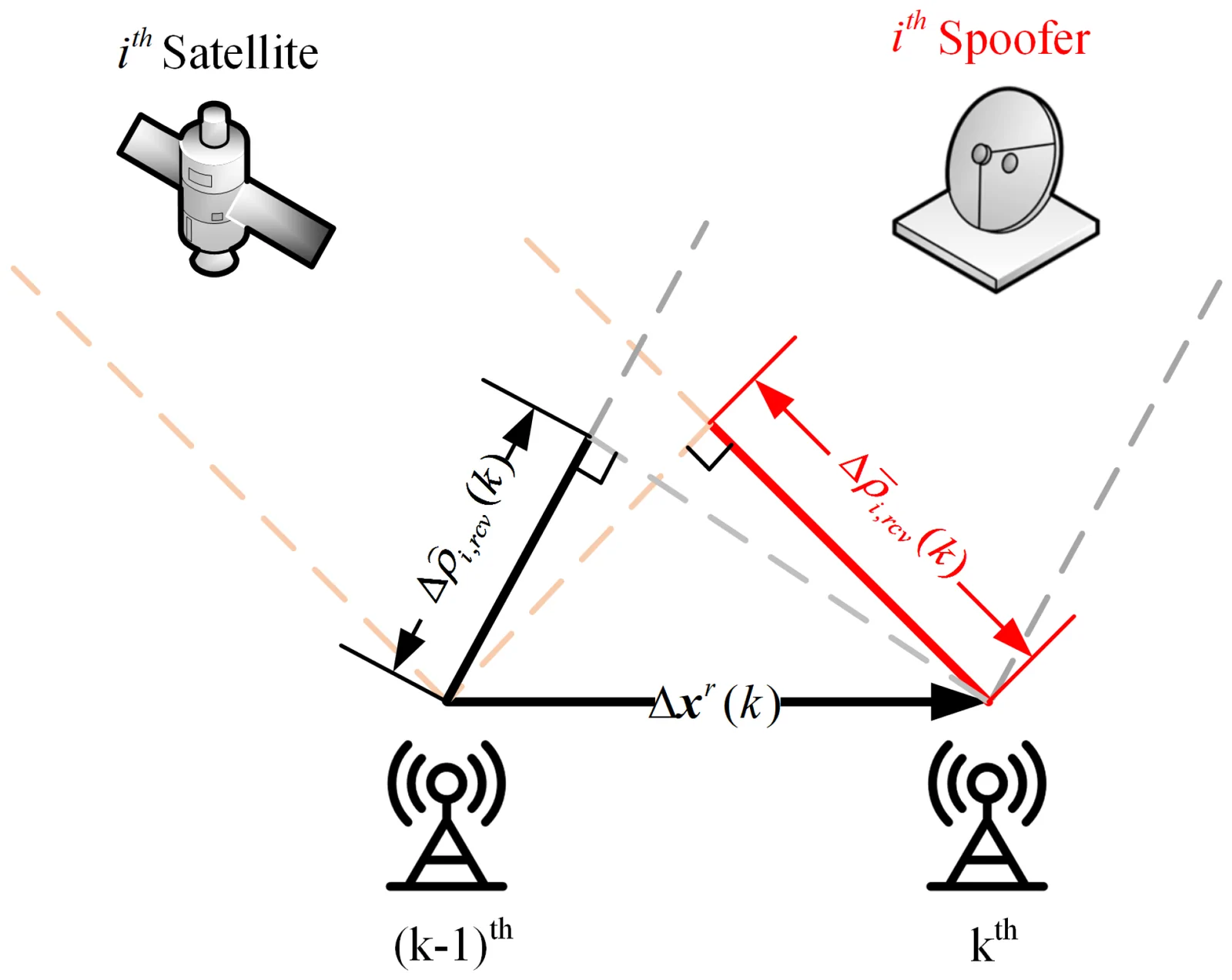
Analysis of the pseudorange difference in Case 3.

**Figure 5:**
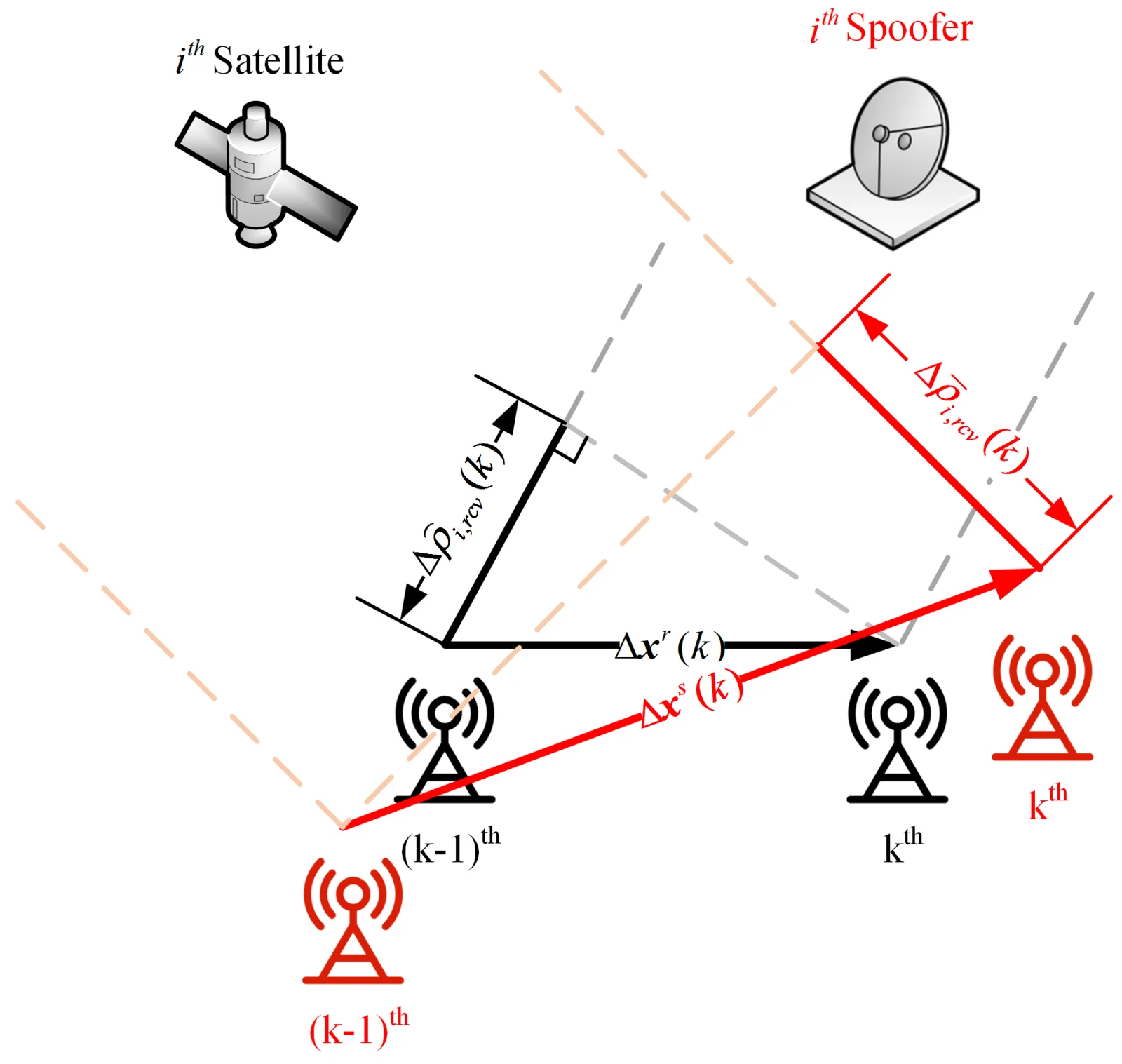
Analysis of the pseudorange difference in Case 4.

**Figure 6:**
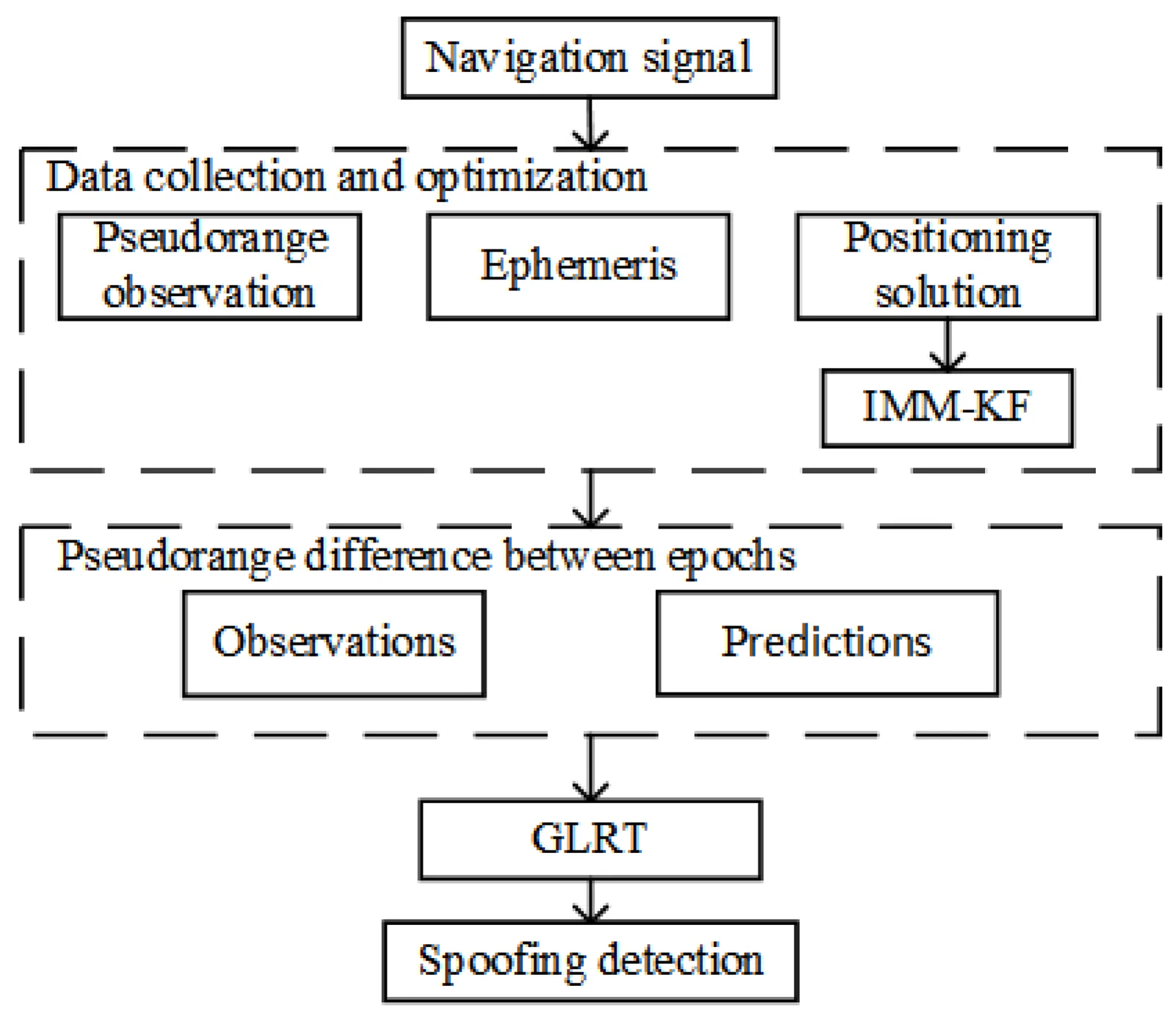
Spoofing detection method architecture.

**Figure 7:**
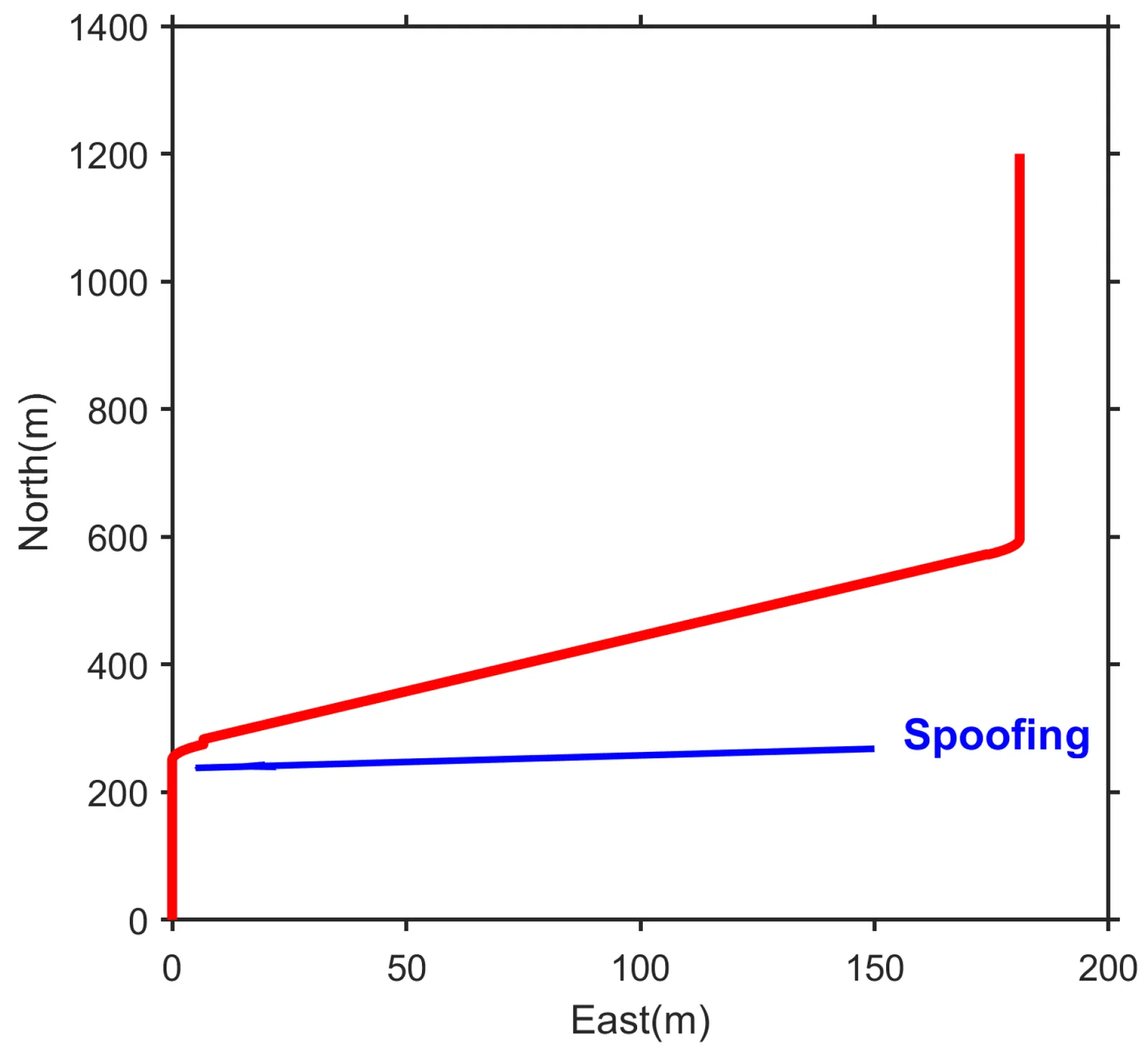
Receiver movement trajectory.

**Figure 8:**
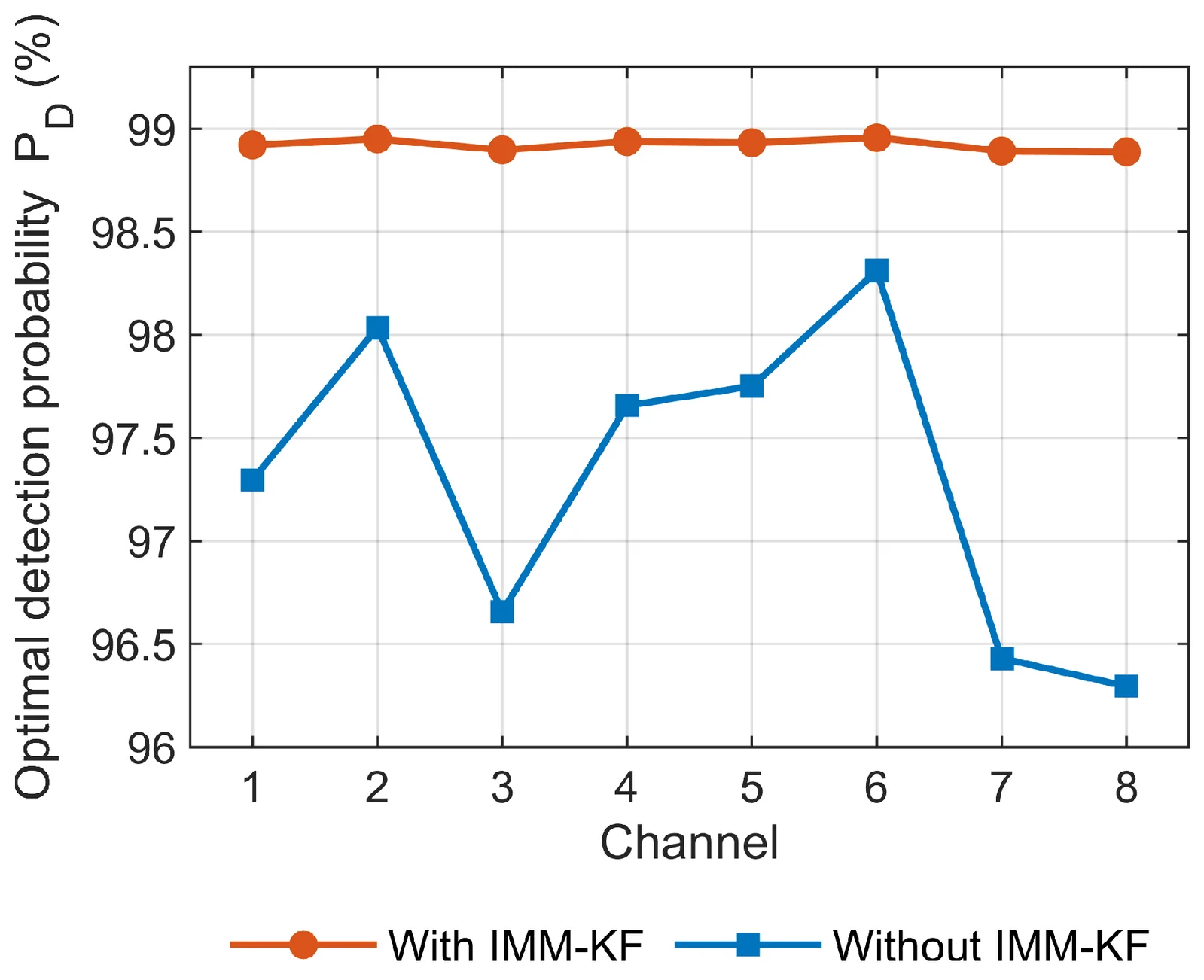
The optimal detection probability of each receiver channel with and without IMM-KF optimisation. The horizontal axis is the receiver channel, numbered 1 to 8, and the vertical axis is the optimal detection probability $P_{D}$ of that channel; the blue squares correspond to the case without the optimisation method and the orange circles to the case with it.

**Figure 9:**
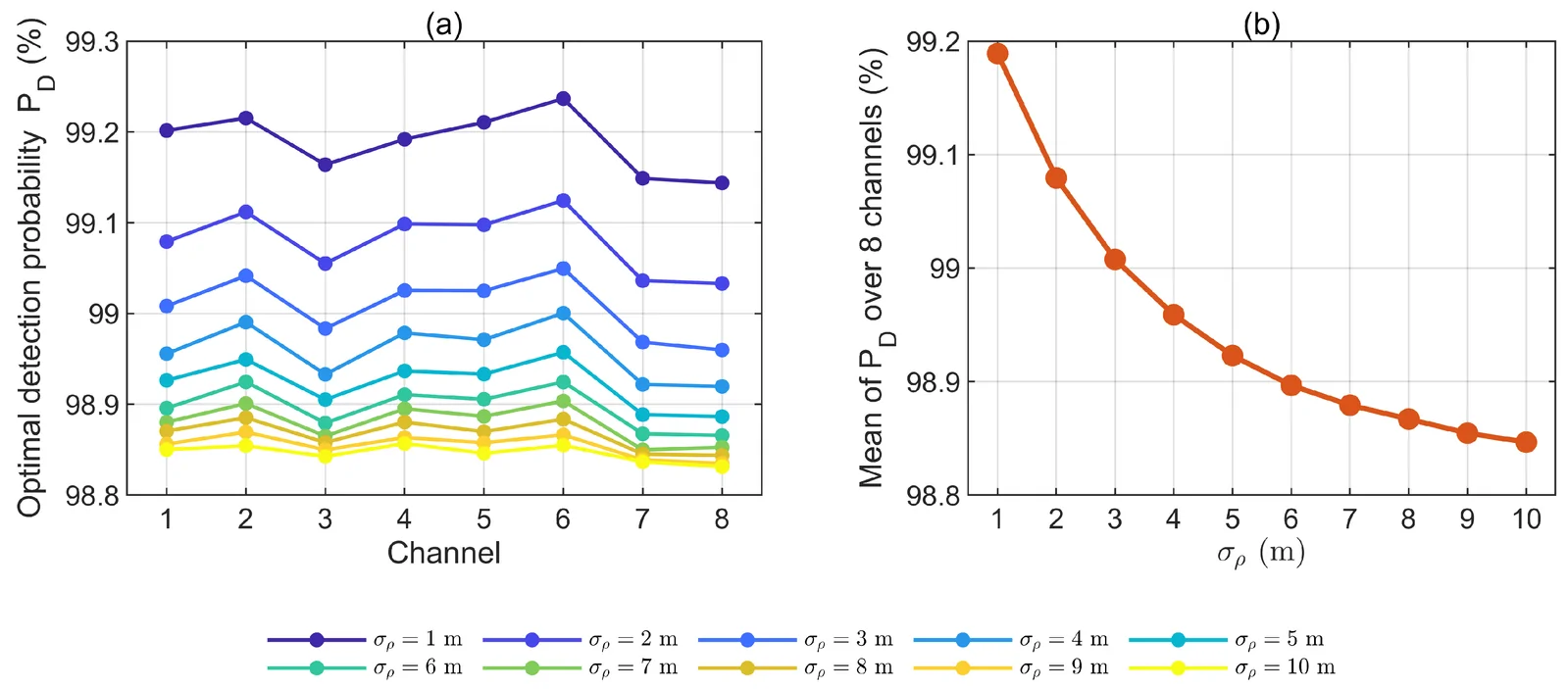
The optimal detection probability with different pseudorange observation errors. (**a**) The optimal detection probability of each receiver channel for every setting of the pseudorange measurement noise $\sigma_{\rho }$; (**b**) the mean over the eight channels at each $\sigma_{\rho }$, every point of (**b**) being the average of the eight curves at the same abscissa of (**a**).

**Figure 10:**
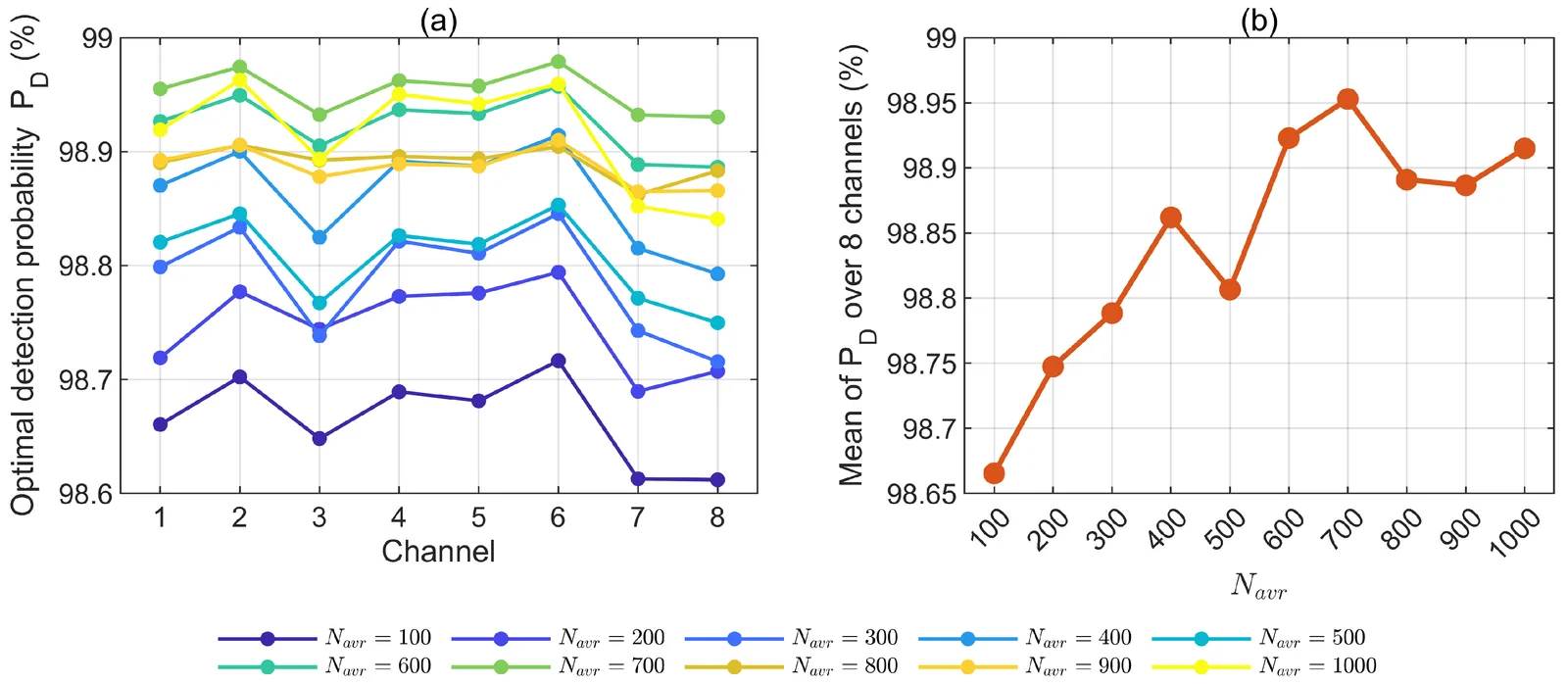
The optimal detection probability with different sample numbers. (**a**) The optimal detection probability of each receiver channel for every length $N_{\mathrm{avr}}$ of the averaging window; (**b**) the mean over the eight channels at each $N_{\mathrm{avr}}$, every point of (**b**) being the average of the eight curves at the same abscissa of (**a**).

**Figure 11:**
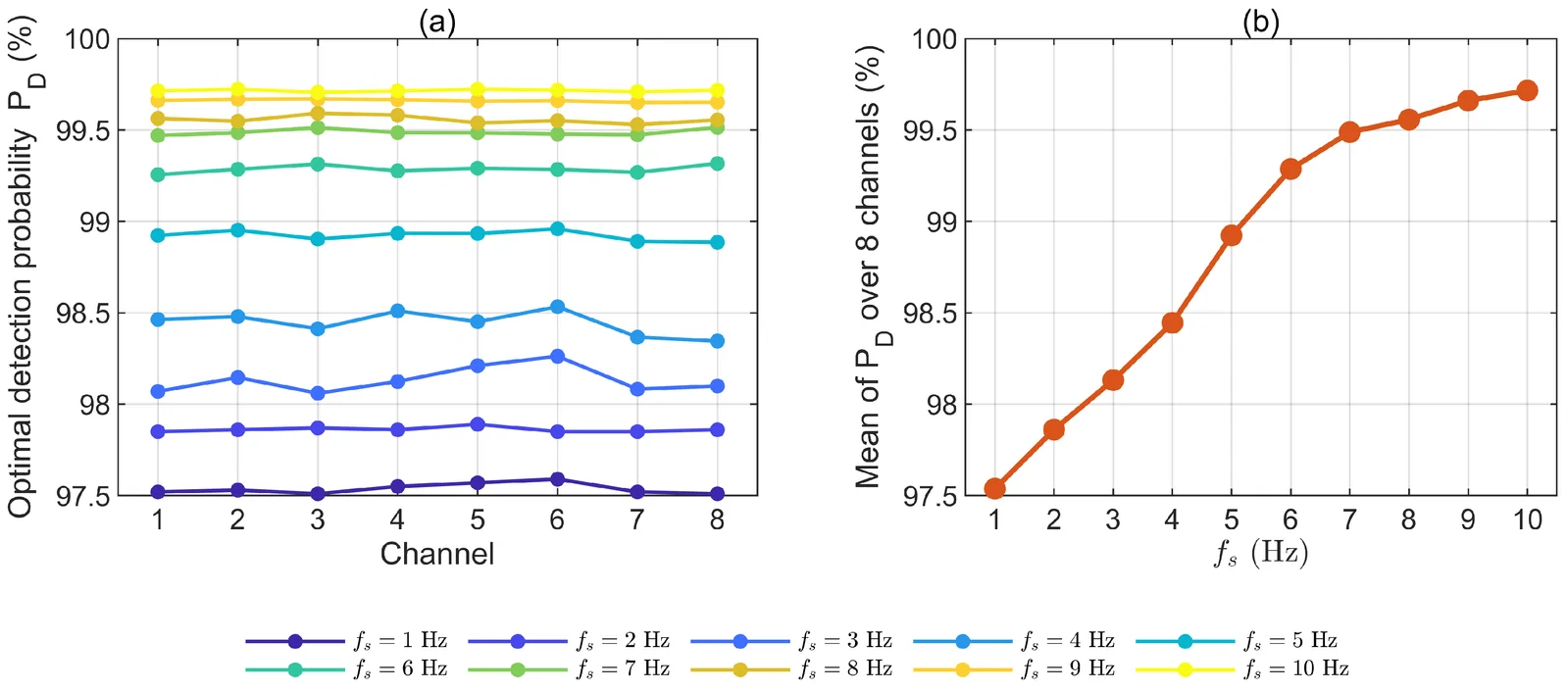
The optimal detection probability with different refresh frequencies. (**a**) The optimal detection probability of each receiver channel for every data refresh frequency $f_{s}$ of the receiver; (**b**) the mean over the eight channels at each $f_{s}$, every point of (**b**) being the average of the eight curves at the same abscissa of (**a**).

**Figure 12:**
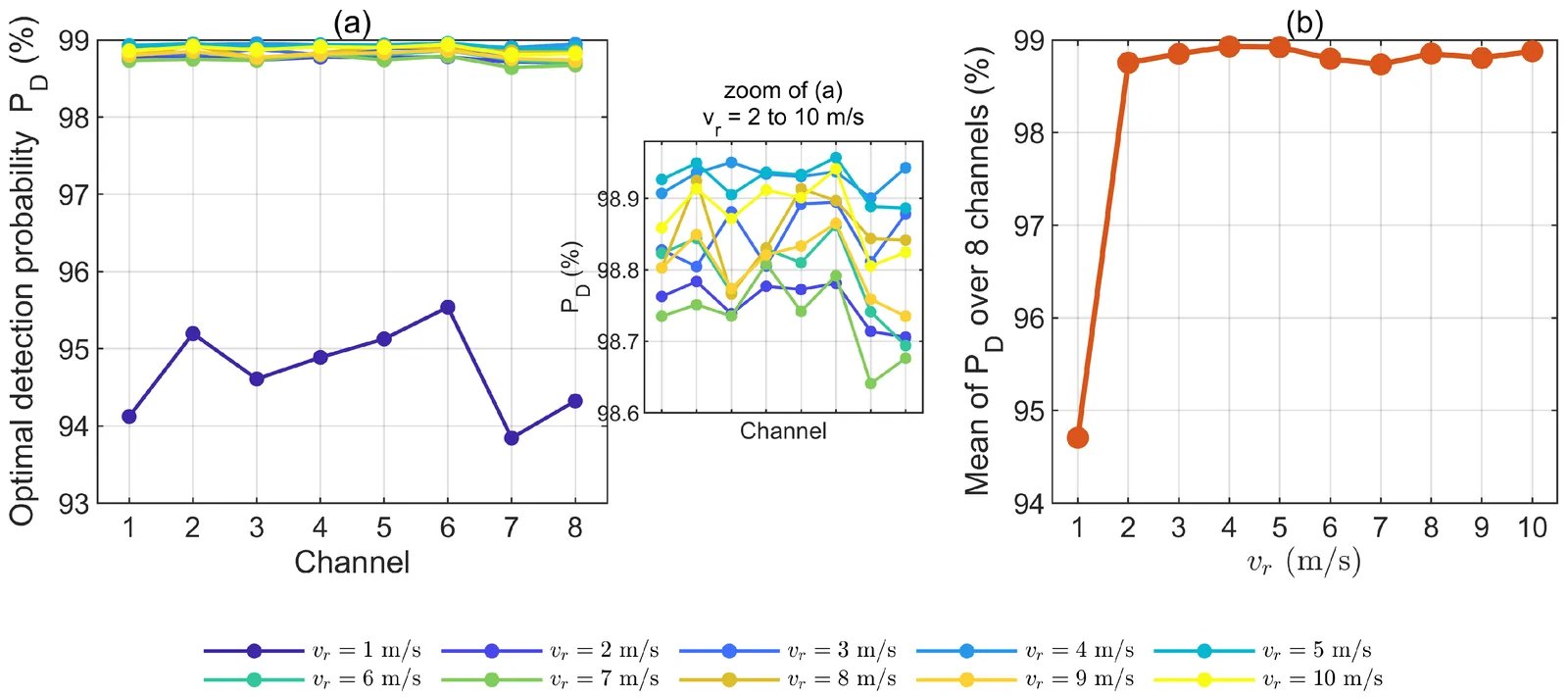
The optimal detection probability with different receiver speeds. (**a**) The optimal detection probability of each receiver channel for every receiver speed $v_{r}$, with an inset enlarging the nine curves that correspond to $v_{r}\geq 2$m/s; (**b**) the mean over the eight channels at each $v_{r}$, every point of (**b**) being the average of the eight curves at the same abscissa of (**a**).

**Figure 13:**
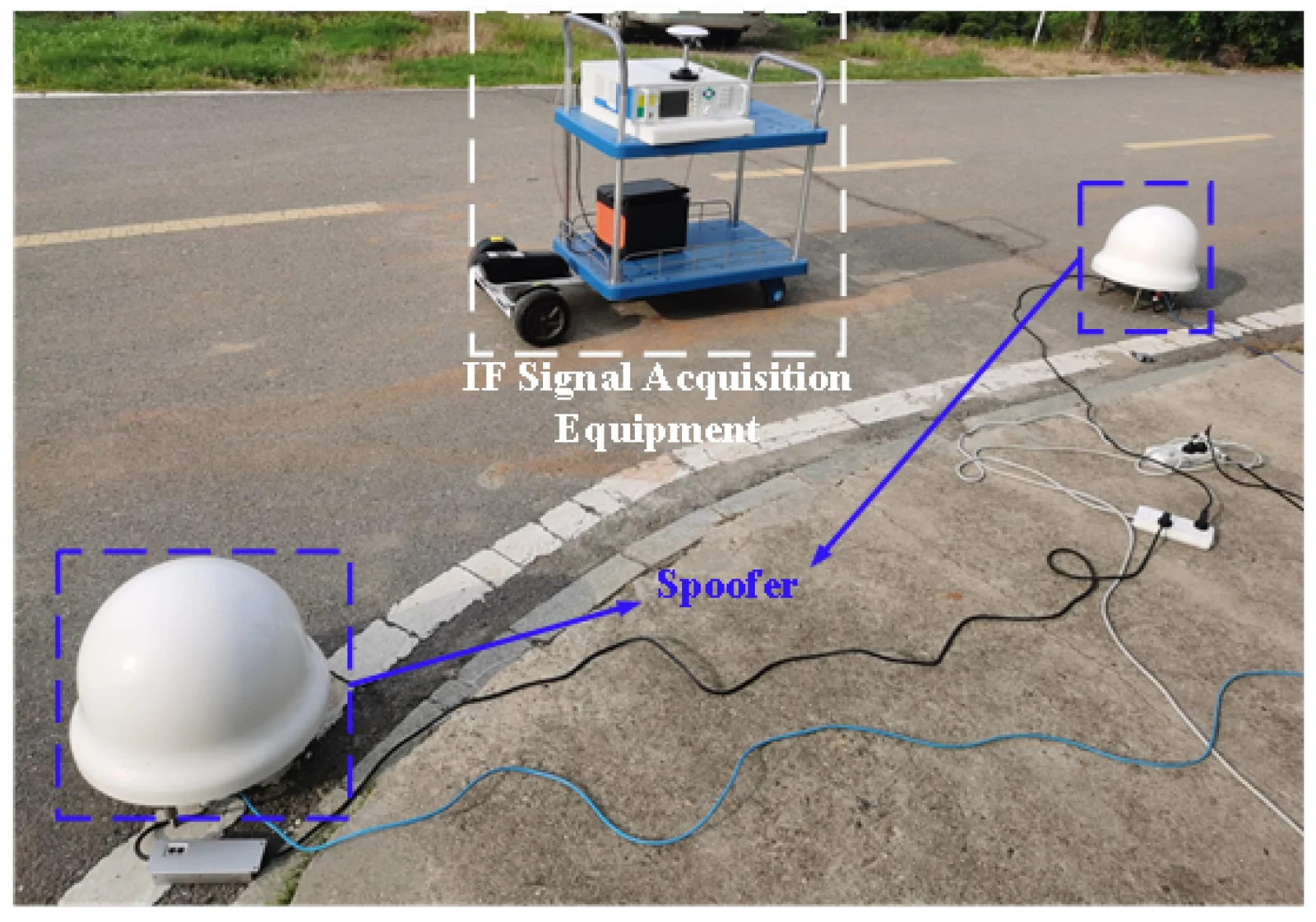
Spoofing scenario.

**Figure 14:**
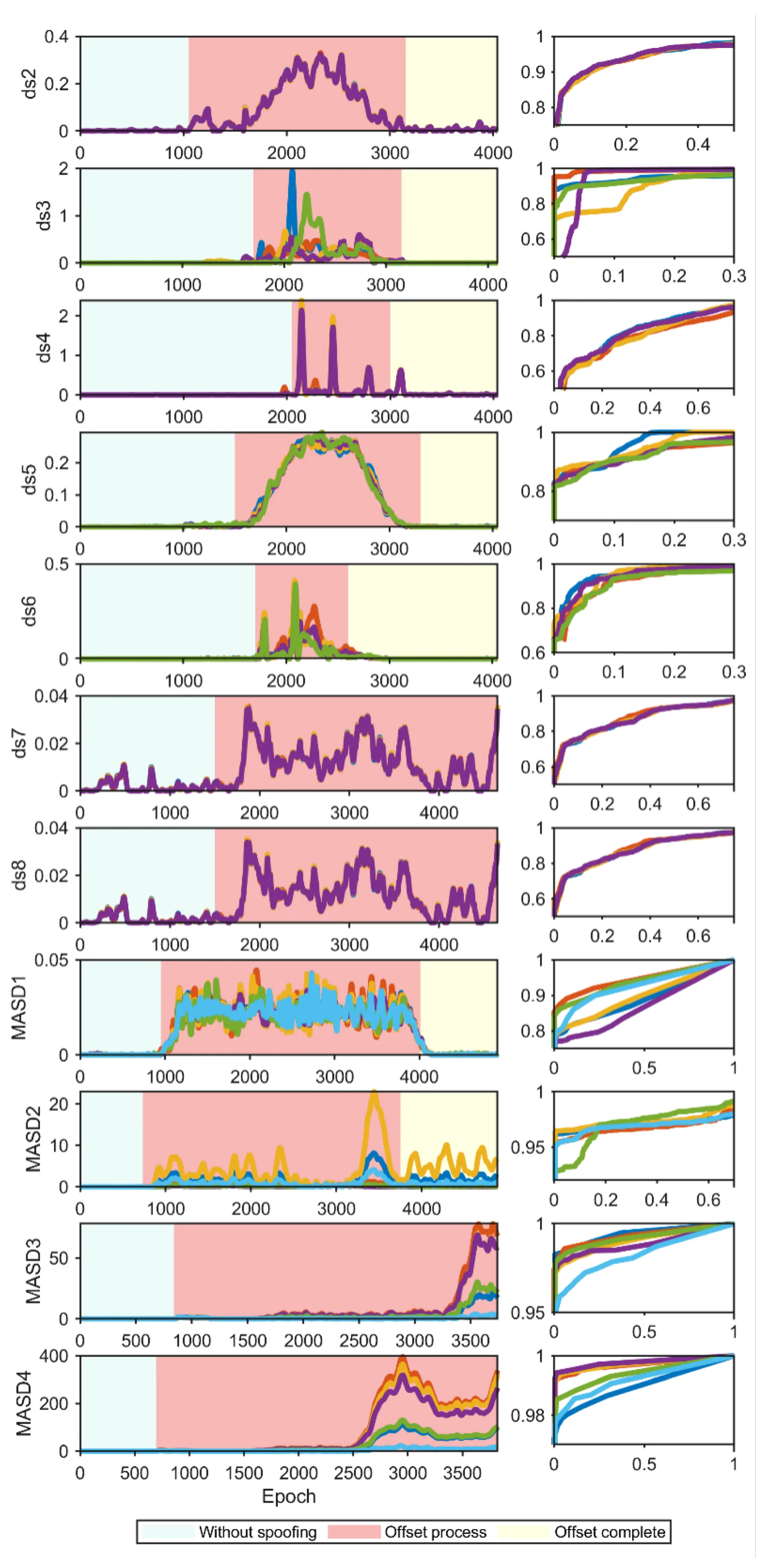
Detection statistics and the corresponding ROC curves for several channels and datasets (sample size 300, IMM-KF enabled). Each of the eleven rows corresponds to one dataset (ds2–ds8 of TEXBAT and MASD 1–4), whose name is printed at the left of the row. Within a row, the left panel plots the detection statistic $T_{i}$ (vertical axis) against the epoch index (horizontal axis); the three background bands denote, in order, the absence of spoofing, the pull-off stage and the interval after the pull-off has been completed. The right panel is the ROC of the same dataset, whose horizontal axis is the false alarm probability $P_{fa}$ and whose vertical axis is the detection probability $P_{d}$, both defined in (14); each ROC curve is traced by sweeping the decision threshold γ of (13). A given colour identifies the same receiver channel in the statistic panel and in the ROC panel. The legend at the bottom of the figure explains the three background bands.

**Figure 15:**
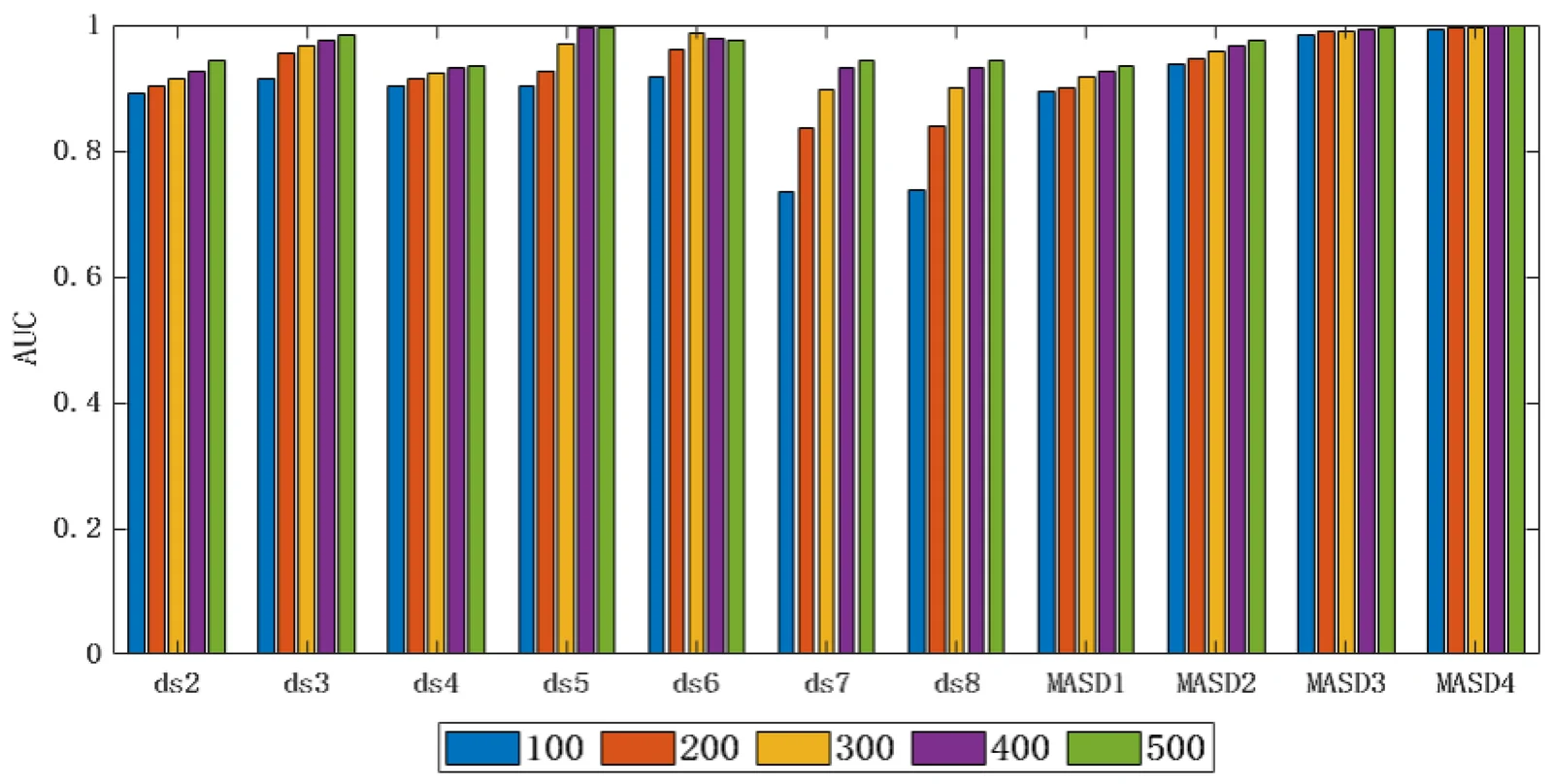
Average AUC values for different datasets and sample sizes. Bar charts of different colors represent different sample sizes. At this point, IMM-KF was used.

**Figure 16:**
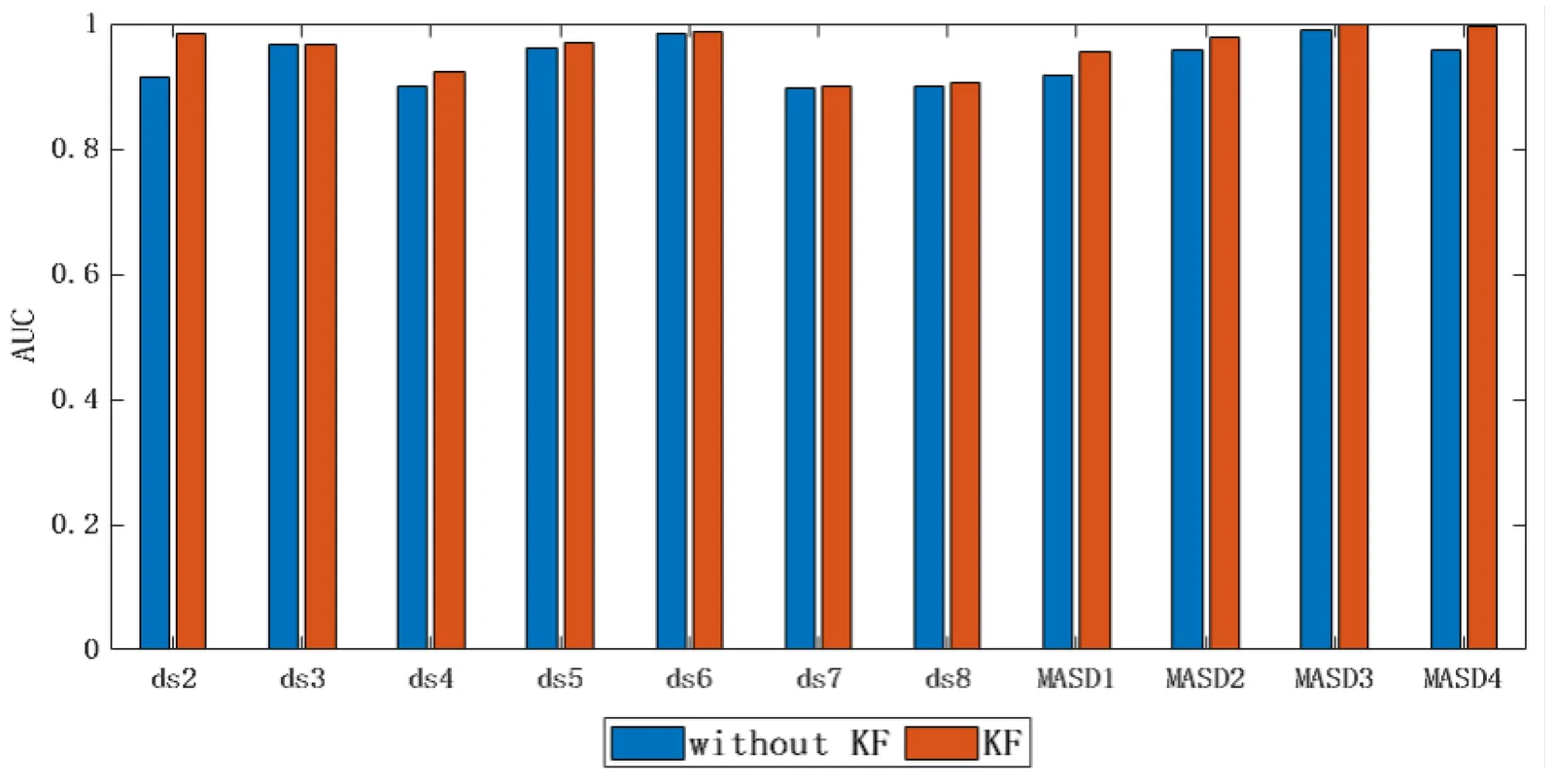
The average AUC of different datasets with or without IMM-KF. The number of samples is 300.

**Table 1:** Introduction to spoofing cases.

Case	Is the Direction of Spoofing Same as Real Signal?	Is the Spoofing Position Same as Real Position?	Possible Types of Spoofing
1	Yes	Yes	Time spoofing
2	Yes	No	Positional spoofing, Time spoofing
3	No	Yes	Time spoofing
4	No	No	Positional spoofing, Time spoofing

**Table 2:** Simulation Parameter Settings.

Parameter	Value
Coordinate of spoofer 1	(100, −100, 100)
Coordinate of spoofer 2	(−100, −100, 100)
Simulation duration	250 s
Spoofer occurrence time	50 s
Number of simulations	100,000
Number of software receiver channels	8
Data refresh frequency of the Receiver	5 Hz
Pseudorange observation error	5 m
Number of samples for the mean method	50
Speed of the Receiver	5 m/s

**Table 3:** Coordinate information of visible satellites.

PRN	X (m)	Y (m)	Z (m)
30	−2,808,543	−21,772,927	14,857,307
7	6,439,590	−25,327,312	3,361,151
28	−7,477,877	−13,710,077	22,116,577
17	−14,633,449	−21,998,228	3,806,262
11	11,869,144	−16,590,391	16,470,689
8	16,191,217	−3,614,163	20,819,819
13	−18,839,646	−5,621,290	17,709,573
1	13,869,630	−20,216,658	9,772,650

**Table 4:** Introduction to TEXBAT.

Dataset	Spoofing Type	Receiver Status	Spoofing Power	Total Duration	Pull-Off Stage
ds2	Time spoofing	Static	10 dB	410 s	110–280 s
ds3	Time spoofing	Static	1.3 dB	410 s	150–275 s
ds4	Location spoofing	Static	0.4 dB	410 s	170–300 s
ds5	Time spoofing	Moving	9.9 dB	410 s	150–270 s
ds6	Location spoofing	Moving	0.8 dB	410 s	150–270 s
ds7	Time spoofing	Static	Gradually increasing	468 s	150–400 s
ds8	Time spoofing	Static	Gradually increasing	468 s	150–400 s

**Table 5:** Introduction to Multi-agent Spoofing Datasets (MASD).

Dataset	Spoofing Type	Receiver Status	Spoofing Power	Total Duration	Pull-Off Stage
MASD 1	Time spoofing	Static	1 dB	500 s	80–380 s
MASD 2	Location spoofing	Static	1 dB	380 s	80–380 s
MASD 3	Time spoofing	Moving	1 dB	500 s	80–380 s
MASD 4	Location spoofing	Moving	1 dB	380 s	80–380 s

## Data Availability

The public TEXBAT dataset used in this study is available from the Radionavigation Laboratory at the University of Texas at Austin. The MASD dataset gathered by the authors is available from the corresponding author upon reasonable request.
